# Molecular Basis for Non-Covalent, Non-Competitive FAAH Inhibition

**DOI:** 10.3390/ijms232415502

**Published:** 2022-12-07

**Authors:** Carmine Marco Morgillo, Antonio Lupia, Alessandro Deplano, Luciano Pirone, Bianca Fiorillo, Emilia Pedone, F. Javier Luque, Valentina Onnis, Federica Moraca, Bruno Catalanotti

**Affiliations:** 1 Department of Pharmacy, University of Naples “Federico II”, Via D. Montesano 49, 80131 Naples, Italy; 2 Net4Science srl, University “Magna Græcia” of Catanzaro, Campus Salvatore Venuta, Viale Europa, 88100 Catanzaro, Italy; 3 Department of Life and Environmental Sciences, Unit of Pharmaceutical, Pharmacological and Nutraceutical Sciences, University of Cagliari, 09042 Monserrato, Italy; 4 Institute of Biostructures and Bioimaging, CNR, 80131 Naples, Italy; 5 Department of Nutrition, Food Science and Gastronomy, Faculty of Pharmacy and Food Sciences, Institute of Biomedicine (IBUB), and Institute of Theoretical and Computational Chemistry (IQTC), University of Barcelona, E-08921 Santa Coloma de Gramenet, Spain

**Keywords:** FAAH inhibitors, propanamide derivatives, molecular dynamics simulations

## Abstract

Fatty acid amide hydrolase (FAAH) plays a key role in the control of cannabinoid signaling and it represents a promising therapeutic strategy for the treatment of a wide range of diseases, including neuropathic pain and chronic inflammation. Starting from kinetics experiments carried out in our previous work for the most potent inhibitor 2-amino-3-chloropyridine amide (TPA14), we have investigated its non-competitive mechanism of action using molecular dynamics, thermodynamic integration and QM-MM/GBSA calculations. The computational studies highlighted the impact of mutations on the receptor binding pockets and elucidated the molecular basis of the non-competitive inhibition mechanism of TPA14, which prevents the endocannabinoid anandamide (AEA) from reaching its pro-active conformation. Our study provides a rationale for the design of non-competitive potent FAAH inhibitors for the treatment of neuropathic pain and chronic inflammation.

## 1. Introduction

Endocannabinoids are endogenous lipid messengers that exert their action by activating a panel of G-protein coupled receptors, including cannabinoid receptors CB1 and CB2 and the transient receptor potential vanilloid 1 (TRPV1) [1]. Although several fatty acid amides have been proposed as endocannabinoids, the two most studied are anandamide (AEA, arachidonoylethanolamide) and 2-arachidonoyl glycerol (2-AG). Both AEA and 2-AG are produced on demand from arachidonic acid and, after their release from the depolarized postsynaptic neurons, they bind and activate the cannabinoid CB_1_ and CB_2_ receptors even with different affinities. Indeed, while AEA preferentially binds to CB_1_, 2-AG activates both CB_1_ and CB_2_ receptors [2]. The endocannabinoid system (ECS) is involved in the regulation of a number of physiological and pathological processes both in the central and peripheral nervous systems, as well as in peripheral organs [3,4].

For this reason, the modulation of ECS has been proposed as a promising therapeutic approach in a wide range of disparate diseases and pathological conditions, ranging from CNS-related pathologies (neuropathic pain, mood and anxiety disorders; movement disorders), to cancer, atherosclerosis, myocardial infarction, hypertension, glaucoma, obesity/metabolic syndrome, and osteoporosis, to name just a few [3]. However, the chronic use of CB_1_ and CB_2_ agonists induces serious adverse side effects for the CNS and the immune systems, respectively [5,6], which limits the clinical utility of direct CB receptor activation [7]. One possible safer mechanism to modulate the ECS is to indirectly activate CB receptors by enhancing the endogenous levels of endocannabinoids AEA and 2-AG, by blocking the processes of their degradation. Enzymatic intracellular degradation of AEA and 2-AG is mediated by the hydrolytic enzymes fatty acid amide hydrolase (FAAH) and monoacylglycerol lipase (MAGL), respectively [8]. An alternative metabolic pathway for endocannabinoids is the direct oxygenation by the type-2 cyclooxygenase (COX-2) into prostanoids, which are lipid mediators of inflammation [9].

**Figure 1:**
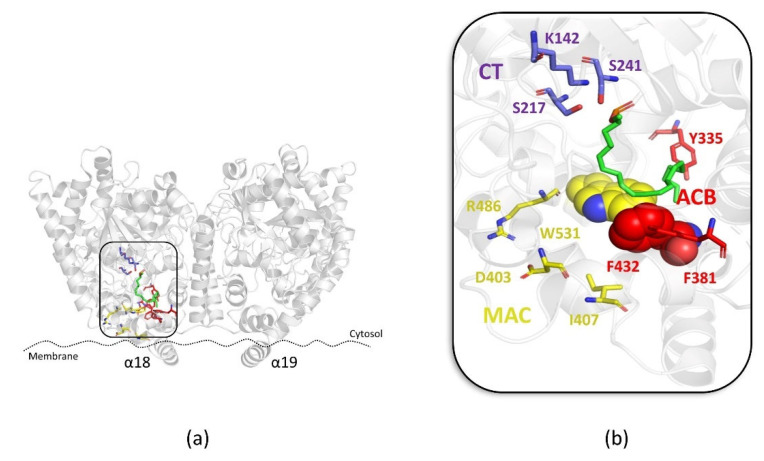
(**a**) Overview of the FAAH protein (PDB: 1MT5); (**b**) zoom view of the binding mode of the AEA derivative FAAH substrate MAFP (green stick) within the “membrane access channel” (MAC) (yellow stick), “acyl-chain binding” channel (ACB) (red stick) and the “catalytic triad” (CT) (violet stick). Residues involved in the “dynamic paddle” are highlighted as space-filling representations.

In particular, during the hydrolysis mechanism of FAAH, AEA reaches the catalytic site through the “membrane access channel” (MAC) delimited by D403, I407, R486, and W531, integrating its arachidonoyl chain into the adjacent “acyl-chain binding” channel (ACB) formed by the Y335, F381, and F432 residues. The polar head of AEA is, instead, oriented toward the K142, S217, and S241 residues that characterized the catalytic region of FAAH, known as the “catalytic triad” (CT). A peculiar aspect of the enzymatic activity of FAAH is the flexibility of the residues located at the boundary between the two MAC and ACB regions, F432, F381, and W531, that act as a *“dynamic paddle”* by directing AEA within the active site for the hydrolysis mechanism (Figure 1a,b) [10].

FAAH, MAGL and COX-2 inhibition results in an increase in the endocannabinoid levels, thus representing a useful approach to increase the endocannabinoid signal, while avoiding the CNS side effects that characterize the direct activation of CB receptors. In light of this, the development of selective FAAH inhibitors [11], as well as dual FAAH/MAGL [12] and FAAH/COXs inhibitors [11], has been largely explored as a therapeutic strategy for the treatment of several diseases, including neurodegenerative disorders, neuropathic pain, and inflammation [13]. Selective FAAH inhibitors were studied as analgesics, anxiolytics [14,15] and antidepressants [15,16,17] and for different cancer malignancies [18,19]. In the clinical phase, FAAH inhibitors have been shown to be well tolerated, with the exception of BIA10-2474, although its severe side effects have been attributed to its binding to unidentified off-targets, rather than its FAAH mechanism-based toxicity [20]. A well-tolerated selective FAAH inhibitor that was evaluated in Phase 1 and Phase 2 studies of patients with social anxiety disorder is JNJ-42165279 by Janssen Research & Development, LLC [21]. In addition, the azetidine analogue V158866 was introduced in clinical study as a reversible FAAH inhibitor, showing good safety, tolerability, pharmacokinetics, and pharmacodynamics properties in patients with neuropathic pain [22].

**Figure 2:**
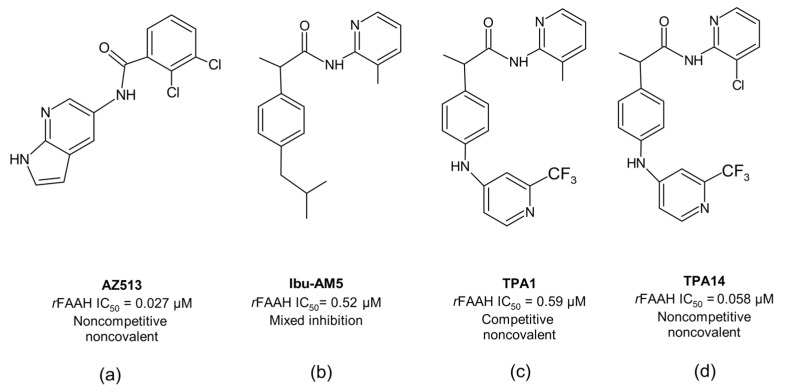
The 2D structures of: (**a**) AZ513; (**b**) Ibu-AM5; (**c**) TPA1 and (**d**) TPA14.

Many different classes of FAAH inhibitors have been reported so far, including competitive and non-competitive inhibitors [23]. The need for long-acting pharmacological activity has mainly been pursued through the search for irreversible inhibitors and, with a few exceptions, such as AZ513 (Figure 2a) [24], the development of non-competitive, non-covalent inhibitors has lagged behind, although they could represent a valid and safer approach to the development of long-acting FAAH inhibitors.

We have recently proposed a new class of propanamide-based FAAH inhibitors, including N-(heteroaryl)-2-(4-((2-(trifluoromethylpyridine-4-yl)amino)phenyl)propanamides, demonstrating how slight modifications in their chemical structure can influence not only the binding mode but also the inhibition mechanism. A clear example is the recently designed 2-amino-3-methylpyridinamide derivative (TPA1) (Figure 2c) and its analogue, bearing the 2-amino-3-chloropyridine ring (TPA14) (Figure 2d). TPA1 was designed from Ibu-AM5 (Figure 2b), by replacing the isobutyl chain with a trifluoromethylpyridinamino moiety. It was observed that this substitution, despite not significantly altering the inhibitory activity against rat FAAH (*r*FAAH) (IC_50_ = 0.52 µM and 0.59 µM for Ibu-AM5 and TPA1, respectively), led to a different inhibition mechanism against *r*FAAH. More interestingly, the substitution of the 3-methylpyridine moiety with the 3-chloropyridine ring of TPA14 not only remarkably increased (by 10-fold) its inhibitory activity with respect to TPA1 against *r*FAAH, but also changed the mechanism of action, from competitive in TPA1 to non-competitive in TPA14 [25]. As for the previously reported AZ513, TPA14 showed no time dependency; therefore, the non-competitive mechanism cannot be due to slow binding. Moreover, incubation experiments have excluded substrate-like activity for this propanamide derivative, and competition experiments between TPA14 and TPA1 have demonstrated that they are mutually exclusive inhibitors. These data lead to the hypothesis that TPA14 binds to the *r*FAAH enzyme at a site that is different to the one identified for TPA1 (Figure 2c). Moreover, we also reported that TPA14 showed reduced inhibitory activity toward the mouse enzyme (*m*FAAH), and interestingly also a different inhibition mechanism, changing from non-competitive to mixed-type inhibition. This information led us to hypothesize that the binding site of TPA14 on *r*FAAH should be in close proximity of the mutating residues from rat and mouse enzymes.

Armed with this information, we have undertaken docking and molecular dynamics (MD) studies, supported by accurate free-energy predictions, of the putative binding mode of TPA14 in *r*FAAH and *m*FAAH. The results are used to discuss a molecular mechanism for the non-competitive inhibition of FAAH.

## 2. Results

### 2.1. Computational Studies

#### 2.1.1. Identification of Putative Binding Sites of TPA14

In order to investigate the binding mode of the non-competitive reversible inhibitor TPA14, we firstly analyzed all the potential cavities in the dimer structure of *r*FAAH (PDB ID: 3QK5) [26] by using the Fpocket webserver [27]. The results of the search were analyzed in light of the different kinetic behavior and potencies of *r*FAAH and *m*FAAH, considering sequence mutations (Supplementary Materials Table S1) and the non-competitive inhibition profile, which indicates that the binding of the substrate AEA is not prevented by TPA14 binding. Fpocket identified many potential binding pockets in the *r*FAAH dimer (Supplementary Materials Table S1 and Figure S1A). Among these, pockets from 8 to 19 were discarded, since they were conserved within *r*FAAH and *m*FAAH sequences. Solvent-exposed pockets 4 and 5 (Supplementary Materials Figure S1D) were also discarded considering the low solubility of TPA derivatives. Pockets 1 and 6 (Supplementary Materials Figure S1B), found in monomers A and B, respectively, identified largely superimposable pockets that contained residues pertaining to ACB and MAC regions. They included the alternative binding site of carprofen (PDB ID: 4DO3) [28] and the urea derivative co-crystallized in PDB ID 3QK5 [26], and presented three mutated residues between *r*FAAH and *m*FAAH (F194Y, I407V, and I530M). Pockets 2, 3, and 7 (Supplementary Materials Table S1 and Figure S1C and S1E, respectively) included the interface between the monomers and embrace residues from the cytosolic port (CP). To the best of our knowledge, the only molecule that binds to this site is the covalent inhibitor OL-135 [29], which partially occupies pocket 2. These pockets also showed mutated residues from rat and mouse enzymes, including G268S and L280V in pocket 7 and S268 in pocket 3. According to the Fpocket results and the information on the enzymatic inhibition profile of TPA14, we considered pockets 1–2 (Supplementary Materials Figure S1B and S1C, respectively), and 3–7 (Supplementary Materials Figure S1C and S1E, respectively) as the most putative druggable pockets for TPA14.

#### 2.1.2. Docking of TPA14 in *r*FAAH and *m*FAAH

We investigated the putative binding of TPA14 in the previously selected pockets 1–2 that contain ACB, MAC and CP channels (Supplementary Materials Figure S2C) and 3–7 (Supplementary Materials Figure S2A,B), through docking calculations followed by molecular dynamics simulations (MDs). Docking calculations for *r*FAAH were carried out on the dimeric X-ray structure with PDB ID: 3QK5 [26], which was successfully used in our previous studies on other propanamide derivatives [25,30], while docking calculations for *m*FAAH were performed using the homology model built as described in the Materials and Methods section. Docking calculations were performed with AutoDock4 ver. 4.2 [31], defining the following two different boxes (box1 and box2), centered in monomer A: (i) box1 includes pocket 1 (Supplementary Materials Figure S2A) and (ii) box2 includes pockets 2, 3, and 7 (Supplementary Materials Figure S2B). Taken together, the two boxes encompassed all the channels present in the FAAH enzyme, from the CP to MAC, passing through the CT and the ACB channel.

Docking calculations in both boxes yielded binding solutions that differed regarding both the ligand’s orientation relative to the CT and position along the channels (Table 1; see also Supplementary Materials Figures S4 and S5, respectively). In the following sections, poses characterized by the proximity of the chloropyridine ring to the catalytic residues (K142, S217, and S241) will be denoted with the letter *A*, while a reversed orientation (i.e., with the trifluoromethylpyridine ring closer to the CT) will be denoted with the letter *B* (Supplementary Materials Figure S3). A third orientation (D-pose) included an area across the MAC and the binding site of the tail of the AEA analogue methyl arachidonyl fluorophosphonate (MAFP) in the PDB structure 1MT5 (Figure 1b) [32]. Both A and B orientations were clustered and classified on the basis of the ligand’s position along the *r*FAAH channels. Accordingly, poses A1 and B1 are in closer contact with the CT, while A3 and B3 positions are located farther from the CT along the MAC channel. Furthermore, the designations “db1” and “db2” are used to define if the A and B solutions were found from docking box1 (db1) (Supplementary Materials Figure S4A) or docking box2 (db2) (Supplementary Materials Figure S5A).

Docking rankings and populations of TPA14 favored poses A2db1 (Supplementary Materials Figure S4C) and B2db1 (Supplementary Materials Figure S4F), which showed a binding mode in the ACB channel, which is very similar to those reported for other profen amides detected in our previous works [25,30,33,34,35]. Nevertheless, the superposition of all the docking poses with MAFP (PDB structure 1MT5) [32] showed a large overlap of poses A1db1, B1db1, A2db1, B2db1, and Ddb1 (Supplementary Materials Figure S4I) on the arachidonoyl chain; therefore, they are unlikely to support a non-competitive mechanism. On the other hand, A3db1 and B3db1 binding poses (Supplementary Materials Figures S4D and S4G, respectively), located in MAC, were in contact with residues F194 and I530, which are Y194 and M530 in the *m*FAAH sequence, respectively. F194, positioned at the gorge of MAC, interacted with the trifluoro-methyl-pyridine ring, while I530, positioned close to the membrane entrance of the channel, interacted with the 2-amino-3-chloropyridine ring. Furthermore, we also found a hydrogen bond (H-bond) of the -NH amino group with residue T488, which was involved in the binding of other propanamides [25,30,34] and carprofen [28].

On the basis of such observations, we selected the A3db1 and B3db1 binding poses on *r*FAAH (Supplementary Materials Figure S4D and S4G, respectively) in order to further investigate the binding mode of TPA14 in the MAC region through molecular dynamics simulations (MDs).

#### 2.1.3. Molecular Dynamics Simulations (MDs) of TPA14 in *r*FAAH

The previously selected docking poses A3db1 and B3db1 (Supplementary Materials Figure S4D and S4G, respectively) were subjected to 100 ns of MD simulations (MDs). Since the docking poses in the MAC area were in close proximity with the membrane, the *r*FAAH enzyme was embedded in a pre-equilibrated 1-palmitoyl-2-oleyl-phosphatidylethanolamine (POPE) membrane. The system was equilibrated during 50 ns of MDs, as described in the Supplementary Materials (Text S1 and Figure S6). MDs were run on the equilibrated structure of *r*FAAH, after loading the A3db1 and B3db1 poses in both monomers A and B (see Materials and Methods section). In all the MDs, the enzyme structure was stable, as demonstrated by the total root mean square deviation (RMSD), which was lower than 3 Å (Supplementary Materials Figures S7B and S7A, respectively).

The RMSD analysis of TPA14 in *r*FAAH, determined with respect to the docking pose, showed a stable binding mode mainly in the MD run of B3db1 (Supplementary Materials Figure S7C), with respect to A3db1 (Supplementary Materials Figure S7D), as confirmed by the cluster analysis (Table 2) and the evolution of key ligand–protein interactions, such as the H-bond with T488 (Supplementary Materials Figure S8A,C,D). In general, RMS fluctuations (RMSF) (Supplementary Materials Figure S9A,B) revealed a few regions characterized by high fluctuations, including helix 1 (residues 7–29), which was manually modelled, and the solvent-exposed loop between helixes 2 and 3 (residues 65–75). Interestingly, in the MDs of the B3db1 binding mode (Supplementary Materials Figure S9B,D), we observed larger fluctuations of residues 390–395, which surrounded TPA14 in monomer B. The cluster analysis reflected the behavior of the RMSD trend; indeed, we found one main prevalent cluster only in the B3db1 trajectories (Table 2).

The most representative clusters obtained from MDs were scored according to the MM/GBSA free-energy method (Table 2). Taking into consideration that clusters from poses A3db1 and B3db1 resided at the interface with the membrane, MM/GBSA calculations were performed on the dry systems that included the same number of POPE units. The best result for each simulation was rescored through QM-MM/GBSA calculations (Table 2), which highlighted the energetic preference for binding to B3db1 in monomer B.

Additional MDs (100 ns) starting from the poses A2db1 and B2db1, which were slightly favored by the docking scores, were performed to compare the free-energy binding determined by using MM/GBSA and QM/MM methods (Supplementary Materials Table S2). The results confirmed that the B3db1 binding mode is the energetically most favored binding mode of TPA14.

**Figure 3:**
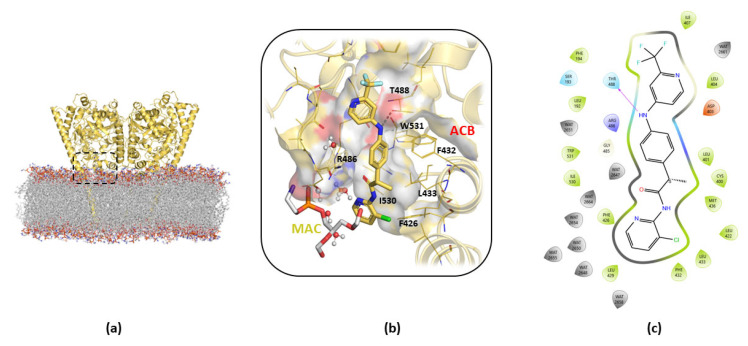
Details of the cluster b0 of the B3db1 binding mode of TPA14 in pocket 1 of *r*FAAH monomer B after 100 ns of MDs. (**a**) Global overview of the TPA14 binding site, indicated by the black dashed rectangle. (**b**) Zoom details of TPA14 (yellow stick) cluster b0 binding mode and its interactions with the surrounding key residues. (**c**) TPA14 2D diagram interaction obtained with Maestro GUI. Magenta arrow indicates the H-bond interaction with T488.

Analysis of the B3db1 binding mode after MDs refinement (Figure 3a–c) revealed that the trifluoro-phenyl moiety of the molecule is located in a hydrophobic pocket, consisting of F194, F381, L401, and L404. The amino -NH group is directly involved in a H-bond interaction with T488 (Figure 3b,c), the pyridine amide carbonyl group that points towards R486 in the MAC channel and the chlorine atom that points towards F426 at the edge of the membrane. In addition, hydrophobic interactions were observed at the gorge of the MAC with I407 and I530 (V407 and M530 in *m*FAAH, respectively).

#### 2.1.4. Non-Competitive Inhibition Mechanism of TPA14

**Figure 4:**
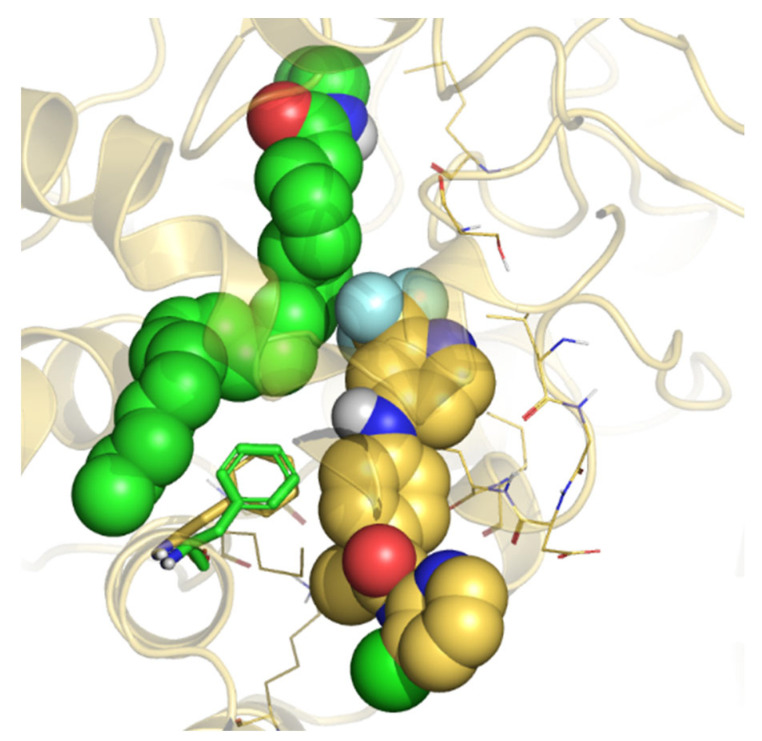
Representation of the 50 ns MD last frame of *r*FAAH in complex with AEA and the B3db1 binding mode of TPA14. AEA and the B3db1 binding of TPA14 are represented as green and gold spheres, respectively. F432 is represented as a green and gold stick for the AEA and TPA14 B3db1 binding simulation, respectively.

According to kinetic experiments [25], TPA14 showed a non-competitive mechanism of inhibition, which means that the binding of TPA14 would prevent AEA hydrolysis, but not the binding itself. Therefore, we performed 100 ns of MDs of the *r*FAAH enzyme in complex with AEA to monitor the existence of AEA conformations that would not be influenced by the binding of the inhibitor (Supplementary Materials Figure S10). The comparison of the clusters obtained from the MDs showed that the main cluster (accounting for 74% in monomer A and 94% in monomer B) was compatible with the non-competitive TPA14 binding mode in MAC (Figure 4).

Moreover, previous MDs studies on the hydrolysis mechanism of FAAH [36] have reported that AEA pro-reactive conformations occur with higher frequency when the tail is located in the MAC channel or in the region located between ACB and MAC; as previously said, F432, in concert with another aromatic residue (W531 or F381), acts as a “dynamic paddle” to regulate the opening of MAC binding channels. This occurs through a change in the dihedral angle between Cα-Cβ (termed φ), thus influencing the dynamics of the acyl chain of the substrate, so that the switch of the φ dihedral angle of F432 from 65° to around 150° controls the access of the AEA tail in the MAC channel. Therefore, the flexibility of F432, and/or of its counterparts W531 or F381, is essential to allow the enzymatic hydrolysis of the substrate. The analysis of the dihedral φ during the MDs of the B3db1 binding mode (Supplementary Materials Figure S11A,C) revealed that TPA14 achieves F432 conformation at an average φ dihedral value of 54°, which is very close to the value of 65° found in the PDB X-ray structure 1MT5 (Figure 1). Interestingly, the F432 dihedrals in the MDs of the binding pose A3db1 (Supplementary Materials Figure S11D), showed a preference for the proactive conformation of F432. No significant changes were observed in W531 or F381 behavior (Supplementary Materials Figure S11). Taken together, these results suggest that the binding of TPA14 to pocket 1 would not interfere with the binding of AEA. However, the conformational flexibility of F432 limits the movements of the AEA tail, impeding the access to pro-reactive conformations, in full agreement with the results of the kinetics experiments that are typical of a non-competitive inhibitor.

#### 2.1.5. Thermodynamic Integration (TI) Calculations

In our previous paper [25], we reported that the energetically most favored binding mode of another TPA derivative, the competitive inhibitor TPA1, was located in the ACB channel (binding mode A1). Nevertheless, the comparative analysis was focused on the binding that resulted from docking; therefore, we did not analyze possible binding modes in the MAC channel. In order to evaluate if TPA1 could bind to the MAC channel, we performed thermodynamic integration calculations (TI) [37] to evaluate the effects of the transformation of the chloropyridine ring in TPA14 to the methylpyridine in TPA1 on the binding affinity in the refined binding mode B3.

The TI results showed that the transition from chlorine to a methyl group resulted in a decrease in free energy of 0.9 Kcal/mol, thus indicating that the binding of TPA1 is disadvantageous at the non-competitive site with respect to TPA14. Moreover, to further prove that TPA1 would bind almost exclusively to the competitive binding site, we simulated a B3 binding mode for TPA1 and performed QM-MM/GBSA calculations. Hence, in order to compare free energies with the previously reported binding mode (namely A1) [25], a 50 ns MD simulation of the A1 binding mode in a membrane-embedded *r*FAAH was run. The results (see Table 3) demonstrated that all the methods used suggested a marked preference for the A1 binding site for TPA1.

Interestingly, the MD simulations of the TPA1 A1 binding mode in an *r*FAAH enzyme embedded in a membrane showed robust and similar binding compared to the published binding mode (data not shown) simulated in the soluble FAAH, thus suggesting that, for binding modes at the competitive site, the approximation of simulations without the membrane is correct.

#### 2.1.6. Molecular Dynamics Simulations (MDs) of TPA14 in *m*FAAH

The differences in the potency and kinetics of inhibition of TPA14 on *m*FAAH with respect to *r*FAAH [25] prompted us to also investigate the binding mode of TPA14 in *m*FAAH. To this end, we performed a docking calculation of TPA14 in the *m*FAAH homology model [25]. The docking calculations showed a marked preference for positions A2db1 (47%) and B2db1 (43%), with residual results in poses A1db1 (4%), B1db1 (4%) and B3db1 (2%) (Table 1). Moreover, since docking in *m*FAAH did not yield any poses that were similar to the A3db1 pose observed in *r*FAAH docking, we manually built the A3db1 pose in *m*FAAH, using as a template the A3db1 pose found in *r*FAAH. We applied the same computational protocol that was adopted before to define the binding mode in *r*FAAH, refining the best ranked docking poses A2db1 and B2db1, and the poses A3db1 and B3db1 relative to the binding in the MAC. The analysis of the MD simulation showed good stability of the protein during the entire simulation in all the systems, with average RMSD values lower than 2.4 Å (Supplementary Materials Figure S12). The cluster analysis (Table 4) revealed the convergence of the TPA14 binding mode in the two monomers in the poses A2db1 and B2db1, as shown by the RMSD superposition of the monomers (Supplementary Materials Table S3), the TPA14 RMSD trend (Supplementary Materials Figure S13), and the superposition of the clusters (Supplementary Materials Figure S14C), while maintaining the H-bond interaction mainly with L192 rather than G485 (Supplementary Materials Figure S14A,B). On the contrary, MDs of A3db1 and B3db1 poses (Supplementary Materials Figure S11B,D) led to different clusters (Supplementary Materials Figure S14I) characterized by an RMSD value around 5 Å in monomer B, implying lower stability for binding modes located in the MAC, mainly due to sequence mutations (I530M and I407V) that reduce the van der Waals contacts with TPA14 and make the gorge of the pocket that faces the membrane wider in *m*FAAH, thus influencing the trend of the key-interactions between TPA14 and *m*FAAH TPA14/*m*FAAH key interaction trend (Supplementary Materials Figure S14D–H). The analysis of the RMSF of the residues suggested that the overall structure is very stable, as found in the MDs of *r*FAAH. The regions mainly involved in the structural rearrangements were the Tm helix (residue 7–29) and the solvent-exposed loops (residues 65–75 and 152–162) (Supplementary Materials Figure S15).

**Figure 5:**
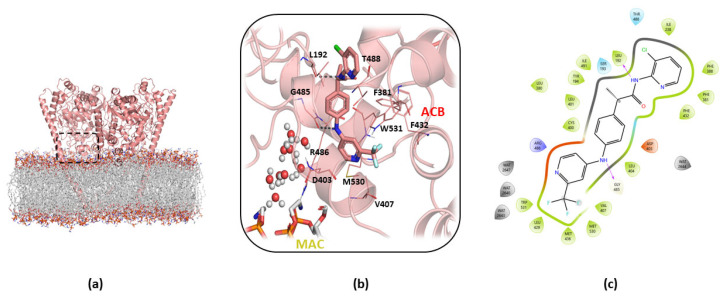
Details of cluster b0 of the A2db1 binding mode of TPA14 in monomer B of *m*FAAH after 150 ns of MDs. (**a**) Global overview of the TPA14 binding site, indicated by the black dashed rectangle. (**b**) Zoom view of TPA14 (pink stick) cluster b0 of the A2db1 binding mode and its interactions with the surrounding key residues. (**c**) TPA14 2D interaction diagram obtained with Maestro GUI. The magenta arrow indicates the H-bond interactions.

The free-energy calculations suggested that the preferred binding mode was the cluster b0 in A2db1 binding (Figure 5a,b), located in the middle of the ACB channel. In particular, the cluster b0 of the A2db1 binding mode is characterized by the presence of a few polar interactions with the backbone of L192 and G485 and the hydrophobic contacts with aromatic residues F381 and F432 in ACB (Figure 5c). In addition, the trifluoromethyl group of TPA14 occupies a hydrophobic cavity formed by W531, L429, and M426 in the MAC.

Taken together, although the docking results were similar for *r*FAAH and *m*FAAH (see Table 1), the MDs performed starting from the docking poses highlighted a different behavior. In *m*FAAH, the favorite binding mode was indeed A2db1 (Figure 5b), located in the middle of the ACB channel, resembling those of previously reported Ibu-AM5 and Flu-AM1 [30], and partially overlapping with the substrate AEA. On the contrary, the B3db1 binding mode, preferred in *r*FAAH, was not favored in contrast to the A2db1 and B2db1 binding modes (Table 3). These differences resulted in good agreement with the experimental findings on the kinetic inhibition profile of TPA14 in *r*FAAH and *m*FAAH (non-competitive and mixed inhibition, respectively).

## 3. Discussion and Conclusions

In the present study, we describe a computational approach to identify the molecular mechanism of action of TPA14, an N-(heteroaryl)-2-(4-((2-(trifluoromethylpyridine-4-yl)amino)phenyl)propenamide derivative previously disclosed as a non-covalent, non-competitive *r*FAAH inhibitor. Docking studies, followed by explicit MDs of the membrane-embedded enzyme, were performed to investigate the possible binding modes in *r*FAAH and *m*FAAH. The analysis of the MDs trajectories, as well as the MM/GBSA and QM-MM/GBSA free-energy calculations, identified the preferred TPA14 binding site in the MAC channel in *r*FAAH and in the ACB channel in *m*FAAH. Moreover, dihedral analysis of F432 in the MDs of *r*FAAH in complex with TPA14 offered an explanation of the role of TPA14 in *r*FAAH, which locked the residue in a conformation that confined the AEA tail to the ACB channel, thus inhibiting enzymatic activity by preventing the AEA conformational flexibility needed to achieve the pro-catalytic conformation. The ability to bind to the MAC channel binding site is finely tuned by slight differences in both the ligand structure and the aminoacidic composition of the FAAH. Indeed, the TI calculations showed that the transformation of the pyridine substituent, from a chlorine atom (TPA14) to methyl group (TPA1), resulted in a decrease in the free-energy binding, and the QM-MM/GBSA calculations confirmed that the preferred binding mode of TPA1 was A1, i.e., in the proximity of the CT. On the other side, minimal differences in the residue composition of the MAC channel in *m*FAAH changed the inhibition mechanism from non-competitive to a mixed-type mechanism. Accordingly, the simulation of TPA14 binding in *m*FAAH showed a preference for an A2-type binding mode, located in the center of the ACB channel, which appears to partially overlap with the substrate. Indeed, mixed-type inhibition kinetics revealed the capability of the inhibitor to bind to the enzyme irrespective of whether the enzyme had already bound to the substrate or not. Therefore, our hypothesis is that TPA14 may have distinct binding sites in *m*FAAH, showing higher affinity for a competitive binding site. The substrate binding would, therefore, alter TPA14’s ability to bind to the competitive site, and so TPA14 could bind to a low affinity, non-competitive site. Hence, we hypothesize that mixed-type inhibition kinetics derives from the possibility that the inhibitor can bind to two binding sites depending on the absence or presence of the substrate. In theory, the obvious next step to take would have been the experimental validation of the computational findings. To this aim, we tested several batches of wild-type and two mutated *r*FAAH expressed in transfected HeLa cells, which were as follows: (i) the FAAH^T488A^ mutant already used in previous studies [25], and (ii) the double mutant FAAH^G268S/L280V^, designed to mimic the mouse CP in *r*FAAH. The results showed a considerable batch variation in the observed potencies of the FAAH inhibitors tested (TPA1, *(R)*-Flu-AM1) [25], which prevents us from making firm conclusions from this approach.

**Figure 6:**
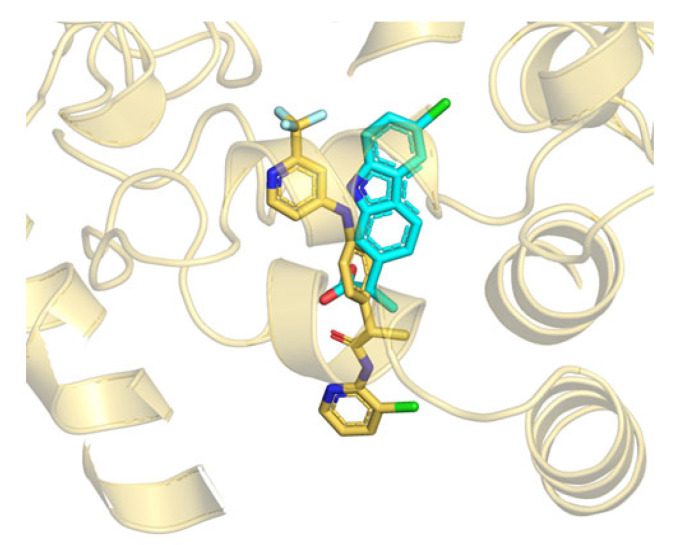
Superposition between the B3db1 binding mode of TPA14 in *r*FAAH (yellow stick) and the crystal structure of carprofen (cyan stick) (PDB: 4DO3), close to the MAC channel.

Taken together, the computational data strongly support the hypothesis that the non-competitive inhibition shown by TPA14 in *r*FAAH may be due to a mechanism that could be explained through the conformational dynamics of the FAAH binding pockets and the subtle differences among different species. Moreover, considering our previous studies on many different propanamide derivatives (ibuprofen and flurbiprofen amides and TPAs), we can speculate that propanamides with different substituents may bind at different locations of the ACB and MAC channel. The first binding site is located at the catalytic triad level and is represented by the TPA1 binding site, and by other covalent inhibitors, such as MAFP and alpha-ketoheterocycle inhibitors [38]. The second binding site is located in the center of the ACB channel, preferred by ibuprofen and flurbiprofen derivatives, and is represented by the pyrrolo-pyridine ligand in the PDB structure 3QK5 [26]. Finally, the third binding site is located in the MAC channel and is represented by TPA14, as well as by the carprofen in the crystal structure 4DO3 (Figure 6) [28].

In conclusion, our study discloses a possible allosteric binding site and provides a rationale for the design of non-competitive potent FAAH inhibitors for the treatment of neuropathic pain and chronic inflammation.

## 4. Materials and Methods

### 4.1. Structural Models

The following two different FAAH sequences have been considered for this study: (*i*) *r*FAAH with the PDB ID 3QK5 [26], after co-crystallized ligand and water removal; (*ii*) mouse FAAH (*m*FAAH). In this last case, given the absence of experimental structures, the homology model of *m*FAAH was built by taking the FAAH mouse amino acid sequence from the Universal Protein Resource (Uniprot) database (http://www.uniprot.org) using the ID O08914 accessed on 7 January 1997 and by building the 3D homology structure using the SwissModel webserver [39]. Specifically, the *r*FAAH PDB structure 3QKV [40] was used as a template (covered sequence: 100%; sequence identity: 91.6%). The global model quality estimation yielded a score of 0.92. In both cases, N-terminus-missing residues, including the transmembrane ΔTM region (residues 7–29), were modeled, assuming a helical shape for the ΔTM region [32]; (iii) the *r*FAAH (PDB code: 1MT5) was used for the simulation of anandamide. In this case, anandamide (AEA) was built by adding an ethanolamine group to the crystallographic pose of the co-crystallized substrate analogue MAFP, as described by Palermo et al. [41]. Each system was preprocessed using the Molprobity webserver [42] that added hydrogens and corrected flips in histidines, asparagines, and glutamines.

### 4.2. Molecular Docking

The Fpocket webserver [43] was used to predict the possible alternative binding pockets. Docking calculations were performed using the software AutoDock4 ver. 4.2 [31]. The 3D structures of TPA1 and TPA14 were built with PyMOL ver. 1.74. Docking was performed on the monomer A of FAAH. The K142 residue was considered in the deprotonated form according to the proposed catalytic mechanism [34]. Rigid docking was performed on the best two pockets predicted, which were as follows: (i) pocket 1 (competitive binding site) using a grid box of 55 × 65 × 60, in order to include the MAC and ACB channels; (ii) pocket 2 using a grid box (44 × 55 × 53), in order to include the cytosolic port (CP). For each docking run, 100 iterations were performed using default parameters of the Lamarckian genetic algorithm (GALS). Results were clustered around the RMSD criterion (cut-off: ≤ 4 Å). The energetically favored poses of the best clusters, according to the AutoDock4 score (ADscore), were loaded for the two monomers of the dimeric form of both *r*FAAH and *m*FAAH, and each system was used as a starting point for theMDs.

### 4.3. Molecular Dynamics Simulations (MDs)

MDs of the AEA–*r*FAAH complex were performed following the scheme of Deplano et al. [25], while AEA partial charges were taken from the work of Palermo et al. [41]. Membrane-embedded MDs were performed to refine the best docking results using the Amber15 package [44]. The *r*FAAH apo structure was inserted into a pre-equilibrated bilayer formed by 493 units of 1-palmitoyl-2-oleyl-phosphatidylethanolamine (POPE) lipids (see Supplementary Materials, Text S1), which is similar to the model used by Bracey et al. [32]. The system was solvated, neutralized and parametrized using the LEaP module of AmberTools15 [44]. The final system contained about 230,000 atoms. The Amber ff14SB force field [45] was used to treat the protein, while the lipid14 force field [46] was used for the POPE units. The general Amber force field (GAFF) [47] was, instead, used to treat the ligands. Specifically, the charge distribution of the ligands was refined using RESP charges [48] fitted to the B3LYP/6-31G(d) electrostatic potential obtained with Gaussian09 [49]. Each complex was minimized using the convergence criterion for the energy gradient set to 0.01 kcal/mol·Å2 in the following four steps, which involve: (*i*) hydrogen atoms in the system (2500 steps of steepest descent and 2500 steps of conjugate gradient); (*ii*) hydrogen atoms, water molecules and counterions (5000 steps of steepest descent and 10,000 steps of conjugate gradient); (*iii*) hydrogen atoms, water molecules, counterions and POPE units (10,000 steps of steepest descent and 10,000 steps of conjugate gradient); (*iv*) finally, the whole system (10,000 steps of steepest descent and 10,000 steps of conjugate gradient). Then, Langevin dynamics [50] was performed at 100 ps to heat the system from 0 K to 100 K at a constant volume, applying weak restraints on the protein and the lipids (force constant of 10 kcal/mol·Å^2^). Thereafter, in order to equilibrate the environment for the FAAH protein, the volume was allowed to change freely by maintaining the constraints on the protein and lipids with the anisotropic Berendsen regulation (1 atm) [51], using a Langevin collision frequency of γ = 1.0 ps^−1^ during the increase in the temperature from 100 K to 300 K at 300 ps, since POPE forms a liquid-crystalline bilayer under these conditions [52,53]. Thus, the restraints on the lipids were gradually released, followed by the gradual relaxation of the restraints on the protein. The equilibration of the whole system took about 50 ns and the system’s properties were analyzed (Supplementary Materials, Text S1). Finally, docking poses were loaded in the two monomers of the *m*FAAH. For the simulation in *m*FAAH, the mouse protein was superposed to the *r*FAAH equilibrated structure. Each system produced an extended trajectory of 100 ns under the following NPT conditions: a time step of 2 fs, using SHAKE for the bonds that contained hydrogen atoms in conjunction with periodic boundary conditions at constant pressure and constant temperature (NPT); particle mesh Ewald (PME) was used to treat all the electrostatic interactions with a cut-off of 9 Å. Pressure was regulated by the anisotropic Berendsen method (1 atm) and temperature was controlled by a Langevin thermostat with a collision frequency of γ = 1.0 ps^−1^, as this method was identified as the most suitable for bilayer treatment in previous work [54]. The structural analysis was performed using in-house scripts and standard codes of AmberTools15. Cluster analysis was performed using the root mean square (RMS) of heavy atoms of the ligand as the distance metric through a hierarchical agglomerative (bottom-up) approach that defined a critical distance ε value of 1.4 Å for the ligand position, with respect to the protein backbone of the FAAH relative monomer. Graphs were plotted with Gnuplot and figures were made with PyMOL 1.74.

### 4.4. Free-Energy Calculations

The relative ΔG for each binding mode of TPA14 described during MDs was evaluated for the dry complex, using the molecular mechanics generalized born surface area (MM/GBSA) [55] on a trajectory window of 5 ns by capturing snapshots every 0.1 ns interval. We used the open source AmberTools15 package (MMPBSA.py) [56] to perform binding free-energy calculations.

### 4.5. QM/MM Calculations

The contribution due to the formation of the ligand–protein complex in the gas phase was determined by QM/MM calculations. To this end, the ligand was treated at the QM level and all the residues within 15 Å of the ligand were traditionally treated, including the closest 10 POPE units for each binding mode. Accordingly, the electrostatic term accounts for the QM interaction (determined at the B3LYP/6-311+G(d,p) level) of the ligand with the set of point charges of the residues included in the MM region. QM/MM calculations were performed for the set of 50 snapshots considered in the MM/GBSA calculations, averaging the electrostatic and van der Waals components of the individual snapshots. The van der Waals term was determined using the 6–12 expression, as implemented in Amber15. The contribution due to the solvation free energies of the complexes (ΔG_sol_) was calculated by MM/GBSA.

### 4.6. Thermodynamic Integration (TI)

The B3db1 binding mode of TPA14 in monomer B of *r*FAAH was used as a starting structure for the free-energy calculation using TI with the pmemd.MPI module of Amber15. The TI simulations of the alchemical transformation from TPA14 into TPA1 used a single-step soft-core potentials approach [36], as previously described [25]. The standard deviation was derived from the asymptotic value of the ΔG calculated in the last 1 ns of each lambda window.

## Figures and Tables

**Table 1:** Docking results of TPA14 in *r*FAAH and *m*FAAH. Capital letters indicate TPA14 orientation concerning the CT: A-pose, amide bond closer to the CT; B-pose, trifluoromethyl-pyridine ring closer to the CT; D-pose binds diagonally with respect to the CT. Numbers indicate the proximity from the catalytic triad; the lower the number, the higher the proximity; db1 and db2 indicate whether the binding solution was retrieved from docking box1 or docking box2, respectively.

Ligand/Species	Box	IC_50_ (µM)	Pose	Cluster Size (%)	ADscore (kcal/mol)
TPA14/rFAAH	1	0.058	A2db1 B2db1 Ddb1 B3db1 A3db1	61 25 9 4 1	−8.9 −8.7 −8.7 −8.2 −8.1
TPA14/rFAAH	2	0.058	A3db2 B2db2 B1db2 A1db2 A2db2 B3db2	55 20 3 4 2 5	−8.7 −8.5 −8.2 −8.0 −7.9 −7.8
TPA14/*m*FAAH	1	0.48	B2db1 A2db1 A1db1 B1db1 B3db1	43 47 4 4 2	−9.3 −8.9 −8.8 −8.5 −8.0

**Table 2:** Refinement of MDs results of TPA14 in *r*FAAH: cluster analysis and free-energy estimation. Data are reported for best clusters in the monomer A (a) and B (b). Time represents the time interval (in ns) used for MM/GBSA and QM/MM free-energy calculations. Free energies are in kcal/mol. n.d. = no data.

Pose	Cluster	Cluster Size (%)	Time (ns)	MM/GBSA	QM-MM/GBSA
A3db1	a0 a1 a2 b0 b1	44.3 31.1 9.6 64.0 25.5	88–93 95–100 73–78 58–63 95–100	−38.8(±0.4) −44.0(±0.4) n.d −41.9(±0.3) −38.2(±0.3)	n.d −52.5 n.d n.d n.d
B3db1	a0 b0	99.7 100	80.5–85.5 95–100	−43.7(±0.4) −49.3(±0.4)	n.d −66.0

**Table 3:** QM/MM calculations for 5 ns of MD simulation after transformation of TPA14 in TPA1 in the B3 binding mode.

TPA1 Pose	QM-MM/GBSA
**A1**	−71.62
**B3**	−61.28

**Table 4:** MD simulation of TPA14 in *m*FAAH: cluster analysis and free-energy estimation. Data are reported for best clusters in the monomers A (a) and B (b). Time represents the time interval (in ns) used for MMGBSA and QM-MM/GBSA free-energy calculations. Free energies are in kcal/mol.

TPA14 Pose	Cluster	Cluster Size (%)	Time (ns)	MM/GBSA	QM-MM/GBSA
A2db1	a0 b0 b1	92.4 58.3 40.3	95–100 95–100 38–43	−42.54 (±0.31) −45.77 (±0.34) −41.08 (±0.35)	n.d. −59.58 n.d
B2db1	a0 b1 b0 b1 b2	64.7 21.8 47.5 41.6 10.6	95–100 27–32 95–100 48–53 5–10	−46.34 (±0.35) −43.98 (±0.39) −42.34 (±0.36) −43.79 (±0.35) −42.3 (±0.31)	−51.97 n.d n.d n.d n.d
A3db1	a0 a1 b0 b1 b2	81.3 11 39.5 27.3 21.3	95–100 29–34 92–95 21–26 47–52	−47.82 (±0.37) −44.26 (0.4) −41.73 (0.32) −39.54 (0.39) −37.83 (0.32)	−45.84 n.d n.d n.d n.d
B3db1	a0 b0 b1	95.5 62 18.5	95–100 95–100 18–23	−48.27 (0.35) −39.01 (0.37) −37.32 (0.28)	−46.98 n.d n.d

## Data Availability

Not applicable.

## Supplementary Materials

The following supporting information can be downloaded at https://www.mdpi.com/article/10.3390/ijms232415502/s1: Table S1: Residue composition, score and color code of putative binding pockets found by Fpocket. Figure S1: Putative binding pockets found by Fpockets Figure S2: Structural differences between rat and mouse FAAH and position of both docking box1 and box2. Figure S3: Schematic representation of the main “A” and “B” binding mode orientations. Figure S4: TPA14 docking poses in box1 of *r*FAAH; Figure S5: TPA14 docking poses in box2 of *r*FAAH; Text S1: Equilibration and properties of the membrane-embedded systems; Figure S6: Membrane properties during system equilibration; Figure S7: Time evolution (100 ns) of the RMSD (Å) of the protein and the ligand TPA14 in monomer A (cyan line) and monomer B (orange line) of *r*FAAH; Figure S8: Structural differences between the most populated clusters of the B3db1 and the less populated A3db1 binding modes of TPA14 in *r*FAAH, with ligand/rFAAH key interaction graphs. Figure S9: Root mean square fluctuation (RMSF) (Å) of *r*FAAH in monomer A and monomer B; Table S2: RMSD of TPA14 in *r*FAAH; Figure S10: Protein and AEA RMSD (Å) trend in monomer A and monomer B of *r*FAAH; Figure S11: Cα-Cβ dihedral angles plots of the “dynamic paddle”, forming residues F381, F432 and W531. Figure S12: *m*FAAH protein RMSD (Å) trend in complex with TPA14; Table S3: Cluster analysis of TPA14 binding mode in *m*FAAH after 100 ns of MDs; Figure S13: TPA14 RMSD (Å) during 100 ns of MDs in *m*FAAH; Figure S14: Structural differences between the most populated clusters of TPA14 in the A2db1 solution in *m*FAAH and the ligand/*m*FAAH key interactions; Figure S15: RMSF (Å) of *m*FAAH in monomer A and monomer B in complex with TPA14. References [44,50,51,52,53] are cited in Supplementary Materials.
