# Supplementary material for: Molecular Basis for Non-Covalent, Non-Competitive FAAH Inhibition
Source: Int J Mol Sci. 2022 Dec 7;23(24):15502. doi: 10.3390/ijms232415502 (PMC9779292; doi:10.3390/ijms232415502)
Supplement: Supplementary file 1 [file ijms-23-15502-s001.zip › ijms-2043576-supplementary.pdf]

Supplementary Materials

for

Molecular Basis for Non-Covalent, Non-Competitive FAAH Inhibition

Carmine Marco Morgillo [1,†,‡], Antonio Lupia [1,2,†], Alessandro Deplano [3], Luciano Pirone [4], Bianca Fiorillo [1] Emilia Pedone [4], F. Javier Luque [5], Valentina Onnis [3], Federica Moraca [1,2,*], Bruno Catalanotti [1,*]

1 Department of Pharmacy, University of Naples "Federico II", Via D. Montesano 49, 80131 Naples, Italy
2 Net4Science srl, University "Magna Græcia", Campus Salvatore Venuta, Viale Europa, 88100 Catanzaro, Italy
3 Department of Life and Environmental Sciences, Unit of Pharmaceutical, Pharmacological and Nutraceutical Sciences, University of Cagliari, 09042 Monserrato, Italy
4 Institute of Biostructures and Bioimaging, CNR, 80131 Naples, Italy
5 Department of Nutrition, Food Science and Gastronomy, Faculty of Pharmacy and Food Sciences, Institute of Biomedicine (IBUB), and Institute of Theoretical and Computational Chemistry (IQTC), University of Barcelona, E-08921 Santa Coloma de Gramenet, Spain
† These authors contributed equally.
‡ Current address: Astex Pharmaceuticals, 436 Cambridge Science Park, Cambridge CB4 0QA, UK.

Table S1. Residue composition, score and color code of potential binding pockets found by Fpocket. Residues in *r*FAAH that are not conserved in *m*FAAH are highlighted in bold, while residues that are shared between equivalent pockets are underlined.

Pocket	Color code	Score	Chain	Residue *r*FAAH[a]	*m*FAAH
1	Light-Green	38.95	A	K142, S190, M191, L192, S193, **F194**, G216, T236, N237, I238, G239, G240, S241, F244, K263, L266, G268, C269, V270, Y271, G272, Q273, T274, V276, Q277, L278, Y335, L372, S376, A377, L380, F381, D383, F388, C400, L401, D403, L404, **I407**, F432, M436, P484, G485, R486, T488, I491, S492, V495, **I530**, W531, I533.	**Y194, V407, M530**
6	Dark-Green	7.11	B	M191, L192, S193, **F194**, G216, I238, G239, G240, S241, F244, Y335, L372, S376, A377, L380, F381, C400, L401, D403, L404, **I407**, F432, M436, T488, G489, I491, S492, V495, **I530**, W531, I534.	
2	Magenta	29.08	A	T257, N259, K263, S264, T274, A275, Q277, L305, D306, P307, T308, P310, L375, W445, Q448, H449, I451, Q456, C499, L500, D501, W556	
3	Light-Magenta	21.33	B	C144, M191, L192, T257, N259, S262, K263, S264, G265, L266, K267, **G268**, Y271, G272, Q273, T274, A275, V276, Q277, L278, D306, T308, V309, L380, F388, Q448, I451, E452, R455, Q456, I459, A460, K463, D501, P555, W556.	**S268**
4	Dark-Blue	12.21	A	A44, K47, A50, **S51**, T54, T115, S116, Y117, T119, E143, G149, H150, **D151**, T153, E160, G161, P163, H184, F201	**G51, A151**
5	Light-Blue	10.72	B	A44, K47, Q48, A50, **S51**, T54, T115, S116, Y117, T119, E143, G149, H150, **D151**, T153, E160, H184, F201	
7	Orange	6.58	A, B	(A): L154, L156, L158, N159, K267, **G268**, C269, V270, Y271, **L280**, R386, S387, Q390, N391.<br>(B): E442, W445, K446, H449, E450, M453.	**S268, V280**
8	*n.s*	6.44	A	L75, T76, P78, K103, W208, I225, G226, S227, G228, G250, I251, R285, D286, T511, Q575, K576	
9	*n.s*	5.64	A	S146, H150, S152, L158, D159, M162, P163, S164, K263, S264, G265, L266, K267, Y271	
10	*n.s*	5.42	B	K142, S190, M191, L192, S217, T236, D237, I238, S241, G268, C269, V270, L278	
11	*n.s*	4.68	A	R315, V318, S321, R323, P324, L325, W462, K463, L467, D468, L554, P555, W556, Q557, L560, R563	
12	*n.s*	4.13	A	E29, L30, E31, T32, R34, L158, G384, R386, L389, Q390, F392, K393, G394, L408, R409, L410, P411, S412, K415, P438	
13	*n.s*	4.098	B	E106, V107, G110, T111, N206, W208, K209, S227	
14	*n.s*	3.83	B	L158, C269, V270, Y271, S387, Q390, N391	
15	*n.s*	3.81	A	W208, G250, E348, R352, P508, V509, T510, T511, P546, M572, T573, Q575, K576	

16	*n.s*	3.58	A	T32, G33, R34, Q35, K36, A37, S157, L158, N391, K393, G394, D395	
17	*n.s*	3.57	B	P78, L80, Q81, V83, Q84, D286, V287, E288, S289, Y319, R320, R566, E569, K576, Q577	
18	*n.s*	3.46	B	V59, F62, R63, L64, N66, P67, L69, S71, E72, L75, F98, T99, K103, E106	
19	*n.s*	3.41	A	E373, V374, S419, L420, K423, P427, A430, A431, N434, S435	

n.s: not shown in Figure S1

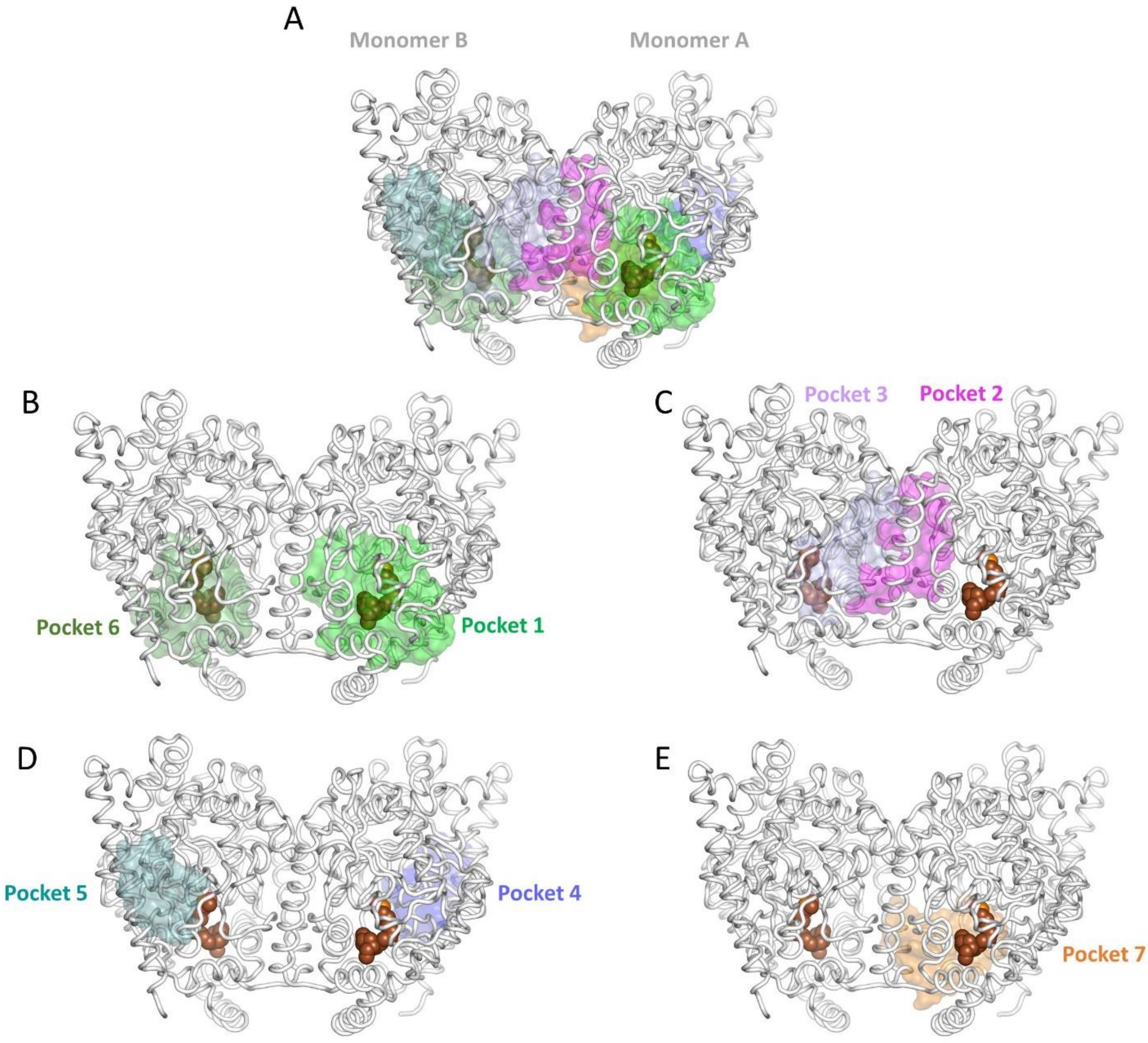

Figure S1. Fpocket results. (A) General overview of the main pockets found by the Fpocket webserver. (B-D) Equivalent pockets depicted as light and dark shades of the same (primary) color code as described in Table S1. (E) Pocket 7 is shown as orange shade. The Anandamide (AEA) analogue MAFP is represented as brown spheres.

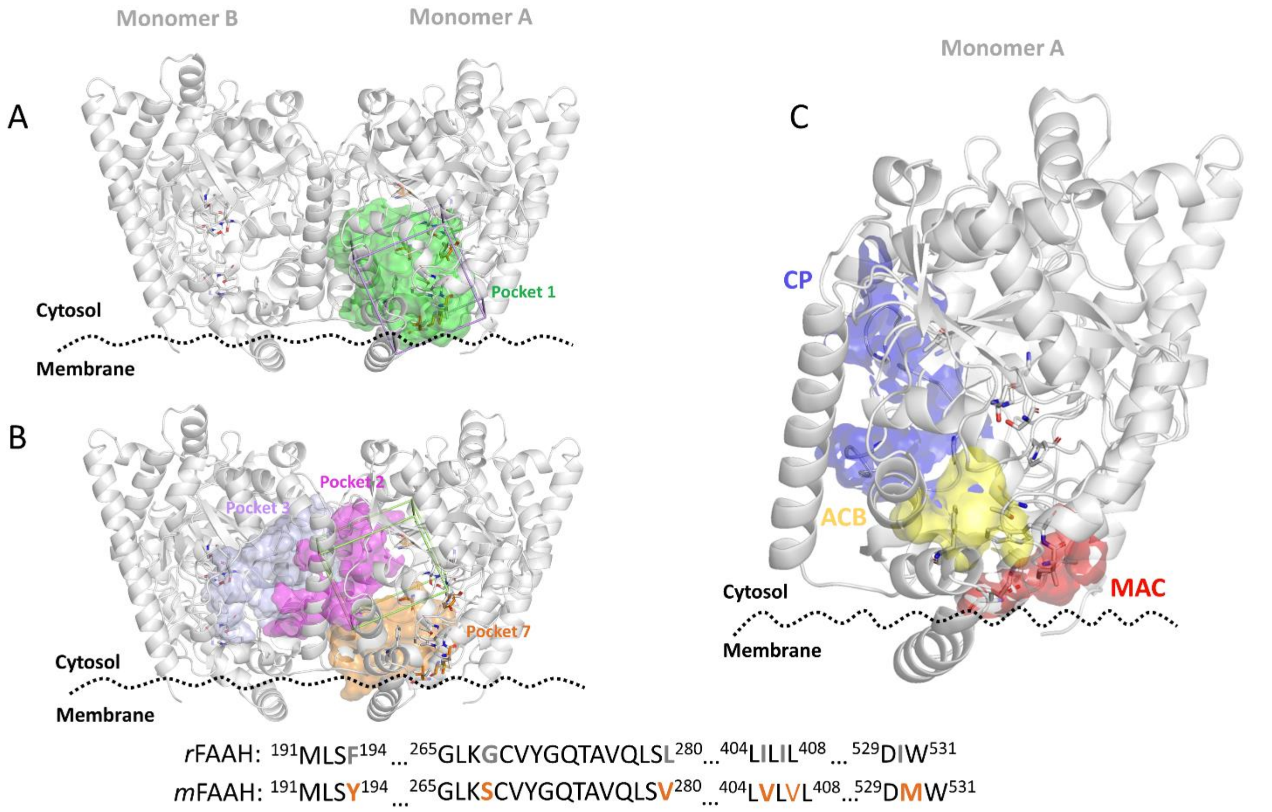

Figure S2. (A) 3D model of *r*FAAH dimer with structural differences with *m*FAAH (orange sticks) in proximity of box 1 embracing residues of pocket 1 found by Fpocket webserver (green surface); (B) 3D model of *r*FAAH defining the box 2 embracing residues of pocket 2, 3 and pocket 7 found by Fpocket webserver (magenta, light-magenta and orange surfaces, respectively). (C) Focus on the monomer A highlighting the CP (blue surface), ACB (yellow surface) and MAC (red surface) regions of *r*FAAH embraced by both box 1 and box 2.

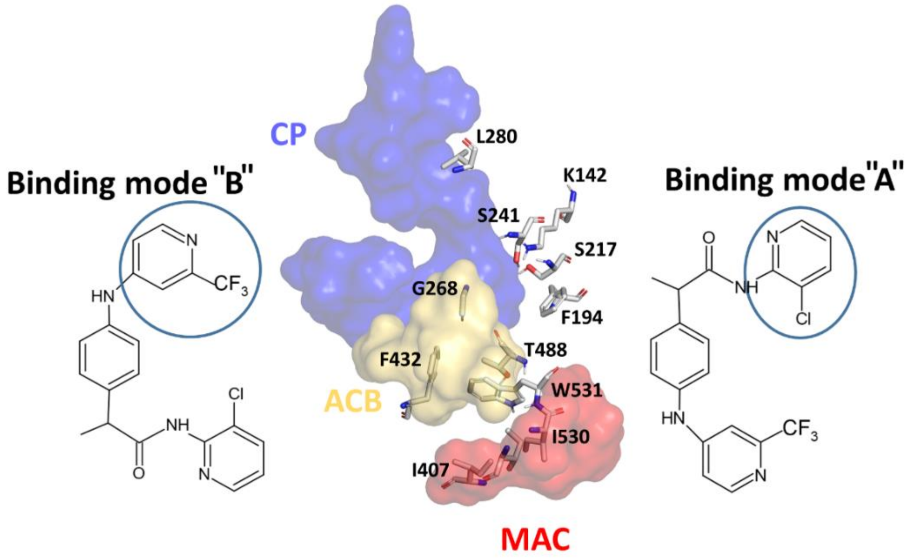

Figure S3. Schematic representation of the main binding mode orientations found by docking calculations: binding mode “A” with the chloropyridine ring to the catalytic residues (K142, S217, and S241), binding mode “B” the trifluoromethylpyridine ring oriented toward the catalytic triad residues.

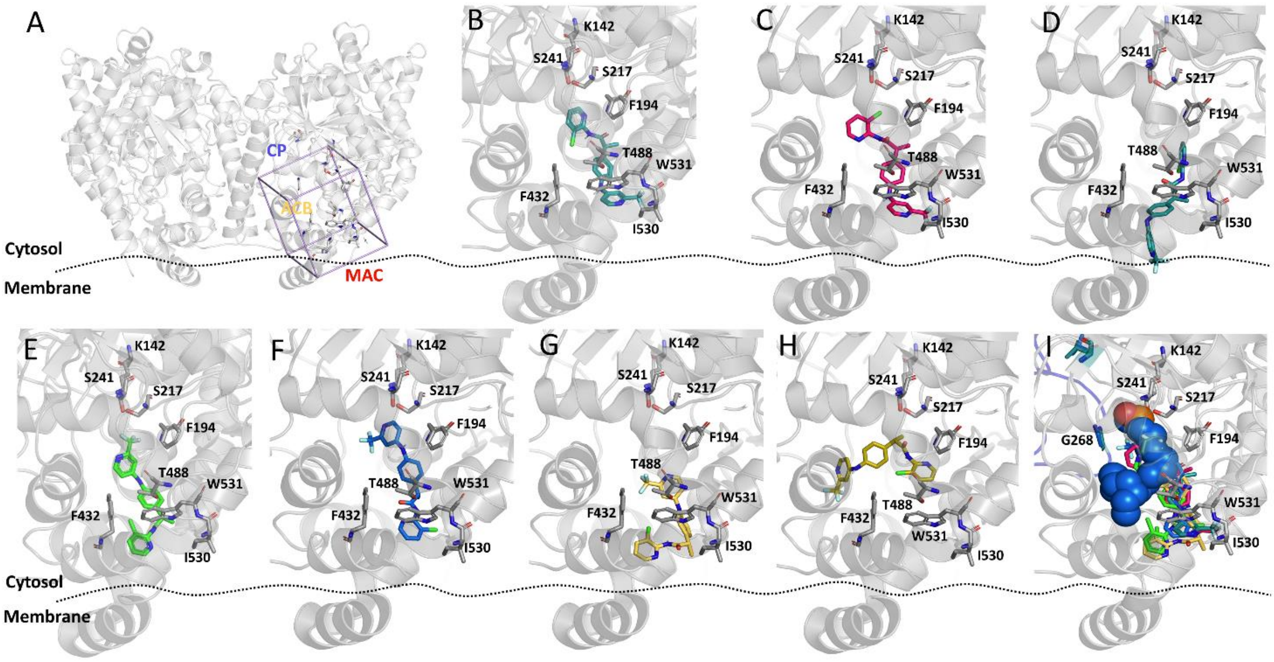

Figure S4. TPA14 docking poses in box 1 of *r*FAAH. (A) Global overview of grid box 1; (B-C-D-E-F-G-H) **TPA14** binding mode A1db1, A2db1, A3db1, B1db1, B2db1, B3db1 and Ddb1, respectively. (I) Superposition between MAFP (blue spheres) (PDB ID: 1MT5) and A1db1, B1db1, A2db1, B2db1, Ddb1 poses of **TPA14**, unlikely to support a non-competitive mechanism. *r*FAAH is showed as grey cartoon.

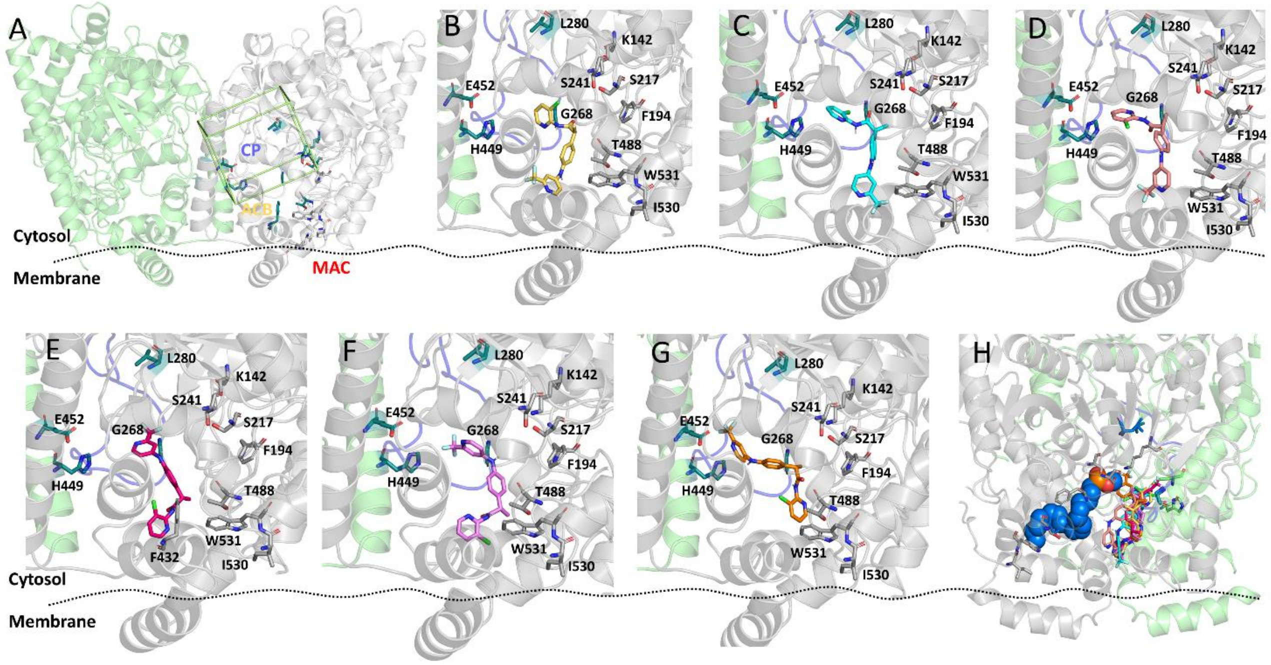

Figure S5. **TPA14** docking poses in box 2 of *r*FAAH. (A) Global overview of grid box 1; (B-C-D-E-F-G) **TPA14** binding mode A1db2, A2db2, A3db2, B1db2, B2db2, B3db2, respectively. (H) Lateral view (90°) of monomer A with the superimposed crystallographic pose of MAFP (blue spheres) highlighting the position of the **TPA14** binding solutions in box 2 with respect to the catalytic site of MAFP. *r*FAAH monomers A and B are showed in grey and green cartoon, respectively; Cytosolic port (CP) is highlighted in blue cartoon. Interacting residues embraced by box 1 are highlighted in grey sticks, while interacting residues included in box 2 are shown as dark-green sticks.

Text S1. Equilibration and properties of the membrane-embedded systems.

*r*FAAH apo dimer, embedded in 493 POPE lipids (about 230000 total atoms), was built using CHARMM Membrane Builder GUI [1]. The system was converted to Lipid14 PDB format using the charmmlipid2amber.x script [2], since an additional layer of TIP3P water molecules were extended, spanning a space of 13 A from the outer edge of the protein along Z axis and counterions were added to neutralize the system using leap module of AmberTools 15 [2]. Langevin dynamics [3] was performed in 100 ps to heat the system from 0 K to 100 K at constant volume, applying weak restraints on the protein and the lipids (force constant of 10 kcal/mol·Å^2). Thereafter, in order to equilibrate the environment for the FAAH protein, the volume was allowed to change freely maintaining the constraints on the protein and lipids with anisotropic Berendsen regulation (1 atm) [4], using a Langevin collision frequency of γ = 1.0 ps^{-1} during the increment of the temperature from 100 K to 300 K in 300 ps, since POPE forms a liquid-crystalline bilayer under these conditions.[5, 6] Thus, the restraints on the lipids were gradually released in 30 ns, followed by a gradual relaxation of the restraints on the protein for a total time of simulation of 50 ns. During this protocol, crucial properties of the system were monitored to confirm that equilibration was reached. The average area per lipid for the POPE molecules was measured during the equilibration (Figure S5) and was determined to be 0.57 nm^2 after 50 ns of equilibration, according with experimental measurement (0.56 nm^2) [7]. Furthermore, an initial large variation of the box dimensions occurred due to a rapid increase of the density of the system, brought by the compression of the system along the XY axis, and the extension along the Z axis as a result of the adjustment of the phospholipids around the transmembrane portion of the protein.

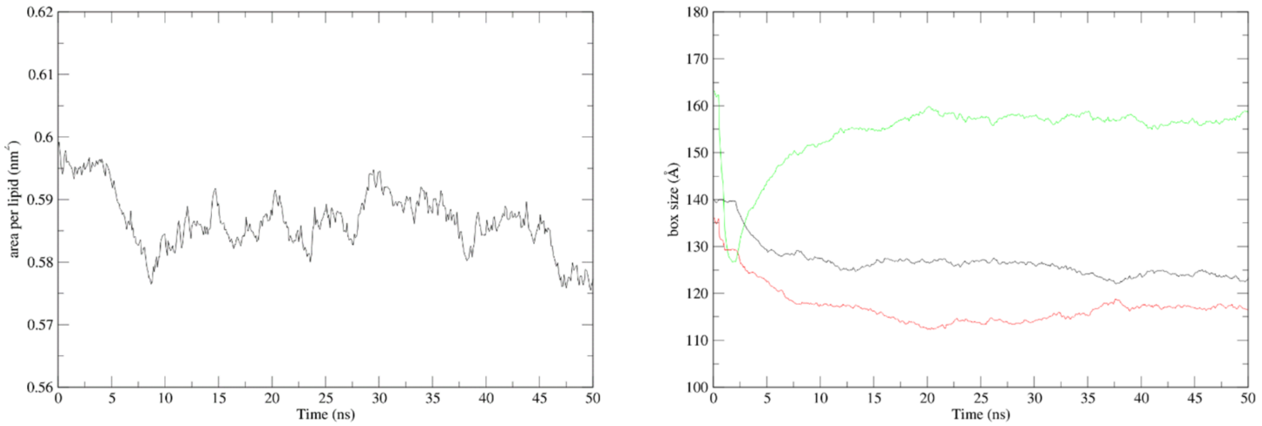

Figure S6. Membrane properties during system equilibration. Left panel: Time evolution of the area per lipid. Right panel: Box size dimensions during the equilibration (X-axis, black; Y-axis, red; Z-axis, green).

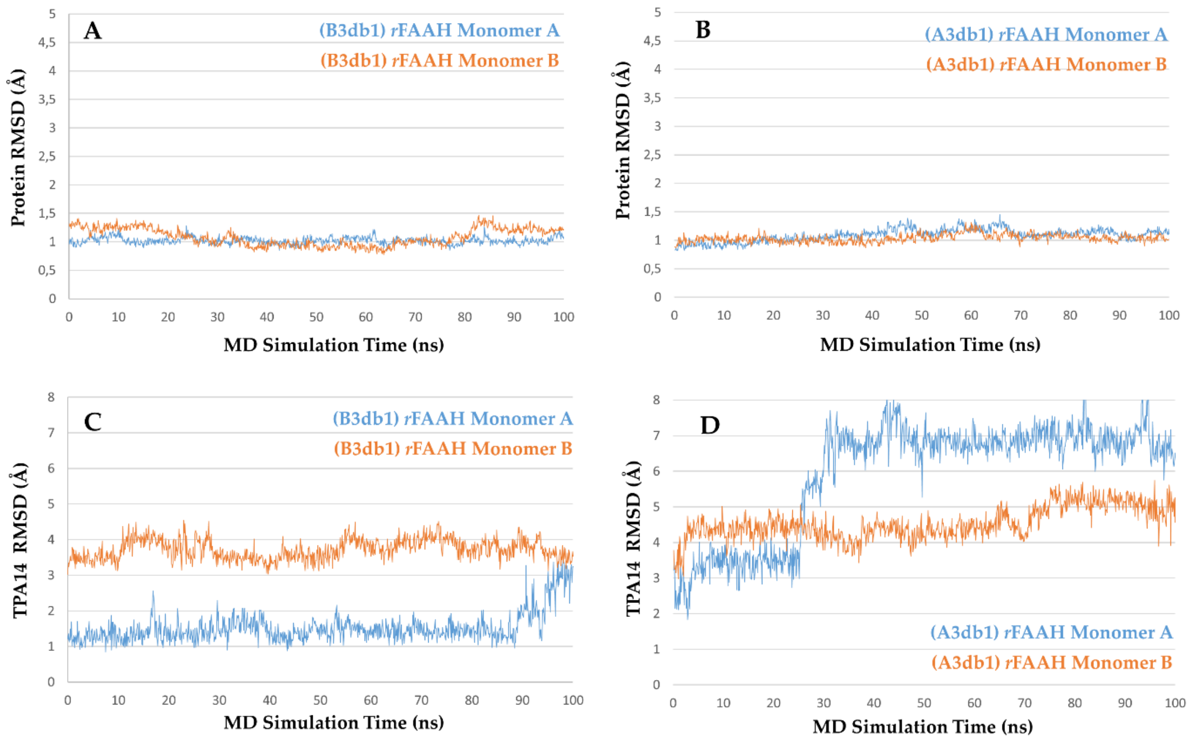

Figure S7. Time evolution (ns) of the RMSD (Å) of the protein and the ligand **TPA14** in monomer A (cyan line) and monomer B (orange line) of *r*FAAH during 100 ns of MDs, with respect to the starting docking poses. (A): protein RMSD of pose B3db1; (B): protein RMSD of pose A3db1; (C): **TPA14** RMSD of pose B3db1; D: ligand RMSD of pose A3db1.

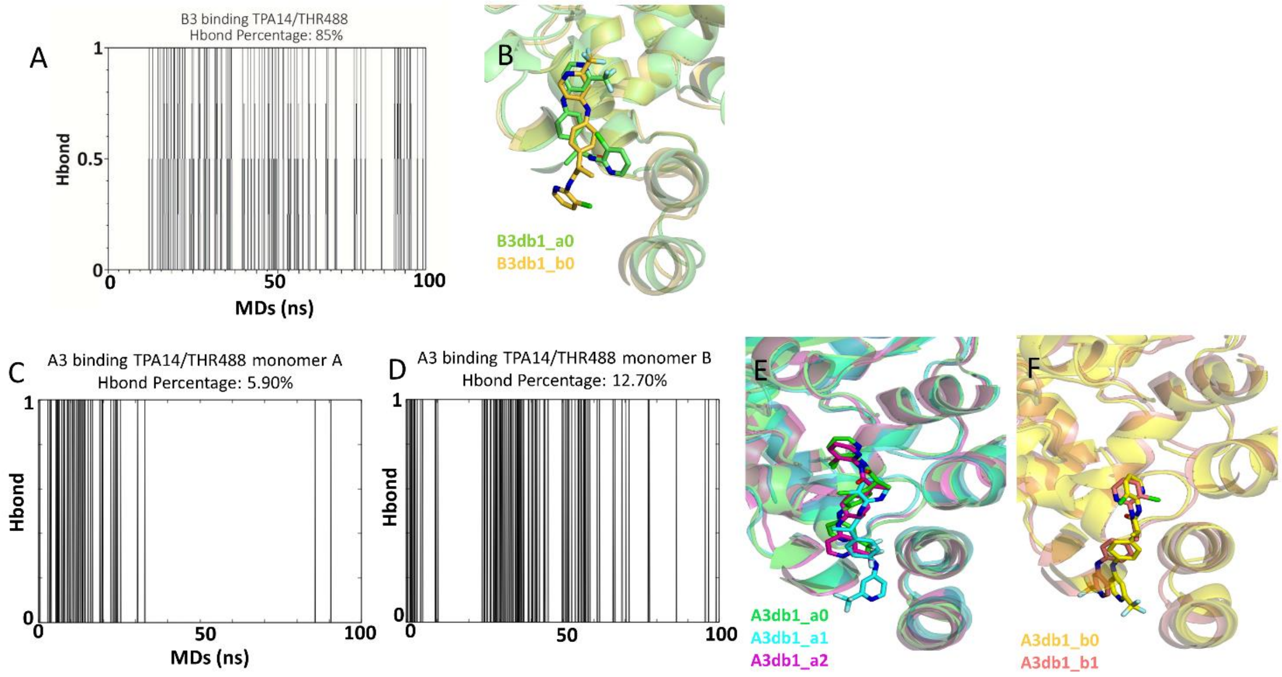

Figure S8: Structural differences between the most stable B3db1 and the less stable A3db1 binding modes of **TPA14** in *r*FAAH. (A) MDs time evolution of the key ligand-protein Hbond between **TPA14**/T488 in the B3db1 binding mode; (B) Superposition of the most populated clusters found in monomer A (a0) and B (b0) of the B3db1 binding; (C-D) MDs time evolution of the key ligand-protein Hbond between **TPA14**/T488 in the A3db1 binding mode in the monomer A and B, respectively. (E-F) Superposition of the most populated clusters found in monomer A (a0, a1, a2) and B (b0, b1) of the A3db1 binding.

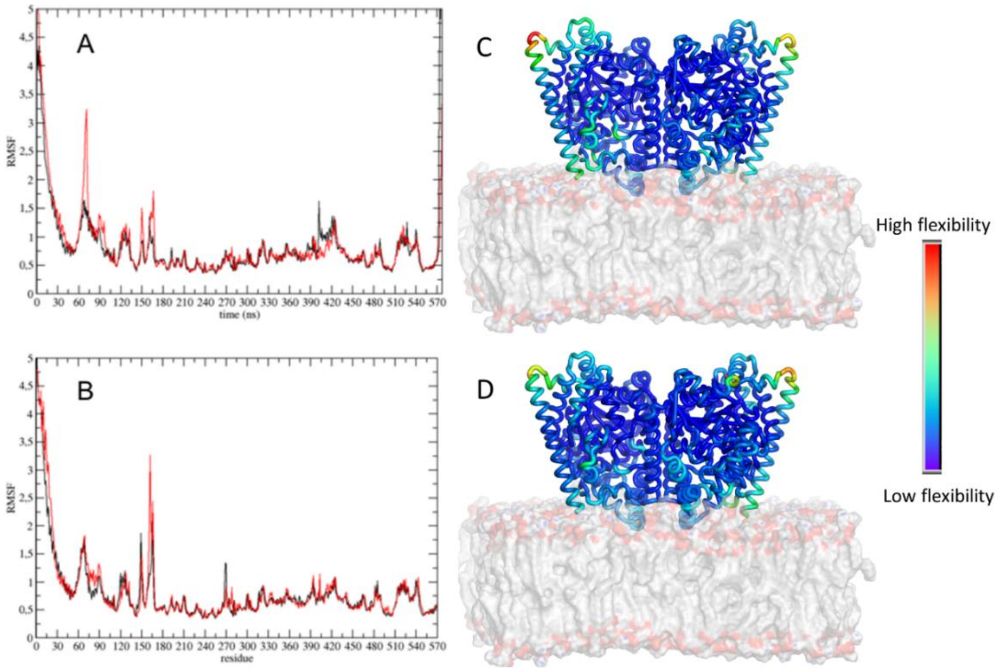

Figure S9: Root mean square fluctuation (RMSF) of *r*FAAH in monomer A (black lines) and monomer B (red lines) during MDs of docking poses (A) A3db1 and (B) B3db1. Putty cartoon representation of the thermal motion RMSF variation in the simulations (C) A3db1 and (D) B3db1: RMSF values are represented on the protein structure within a rainbow colour spectrum from dark blue (lower flexibility) to dark red (higher flexibility).

Table S2. Analysis MD refining results of **TPA14** in *r*FAAH: Cluster analysis and free energy estimation. Data are reported for best clusters in the monomer A (a) and B (b). Time coloumn represents the time interval used for MMGBSA and QM/MM free energy calculations. Free energies are in kcal/mol. (n.d = no data)

Pose	cl.	Pop (%)	Time (ns)	MM/GBSA	QM-MM/GBSA
**A2db1**	a0	95.4	95-100	-46.0 (±0.3)	-55.0
	b0	55	95-100	-41.4 (±0.3)	n.d
	b1	41	40-45	-42.3 (±0.4)	n.d
**A3db1**	a0	44.3	88-93	-38.8 (±0.4)	n.d
	a1	31,1	95-100	-44.0 (±0.4)	-52.5
	a2	9.6	73-78	n.d	n.d
	b0	64	58-63	-41.9 (±0.3)	n.d
	b1	25.5	95-100	-38.2 (±0.3)	n.d
**B2db1**	a0	54.8	95-100	-44.2 (±0.4)	-50.4
	a1	45.2	29-34	-44.0 (±0.4)	n.d
	b0	84.8	59-64	-44.5 (±0.4)	n.d
	b1	14.7	94-99	-43.5 (±0.4)	n.d
**B3db1**	a0	99.7	80.5-85.5	-43.7 (±0.4)	n.d
	b0	100	95-100	-49.3 (±0.4)	-66.0
**Ddb1**	a0	62.3	95-100	-45.3 (±0.4)	-54.1
	a1	35.5	45-50	-45.0 (±0.4)	n.d
	b0	84.2	95-100	-43.0 (±0.3)	n.d
	b1	10.5	6-11	-39.3 (±0.4)	n.d

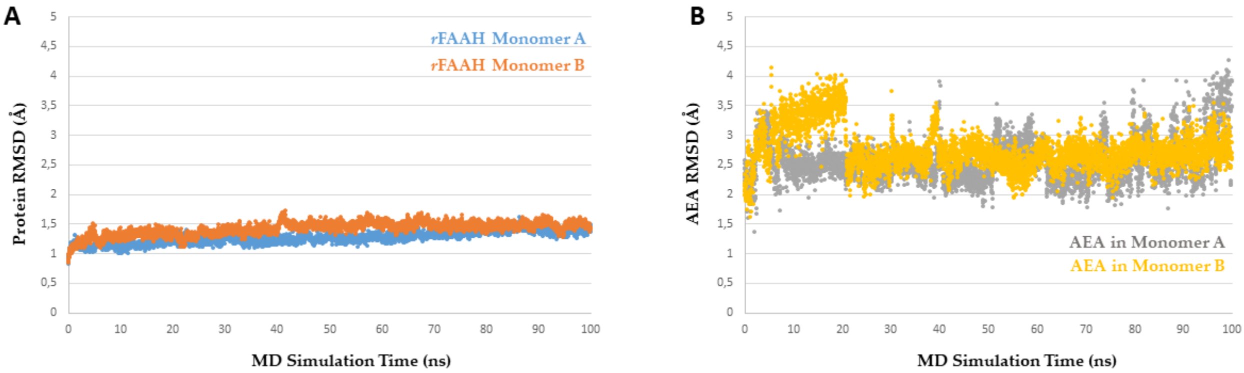

Figure S10: RMSD trend of (A): *r*FAAH and (B) AEA during 100ns of MDs

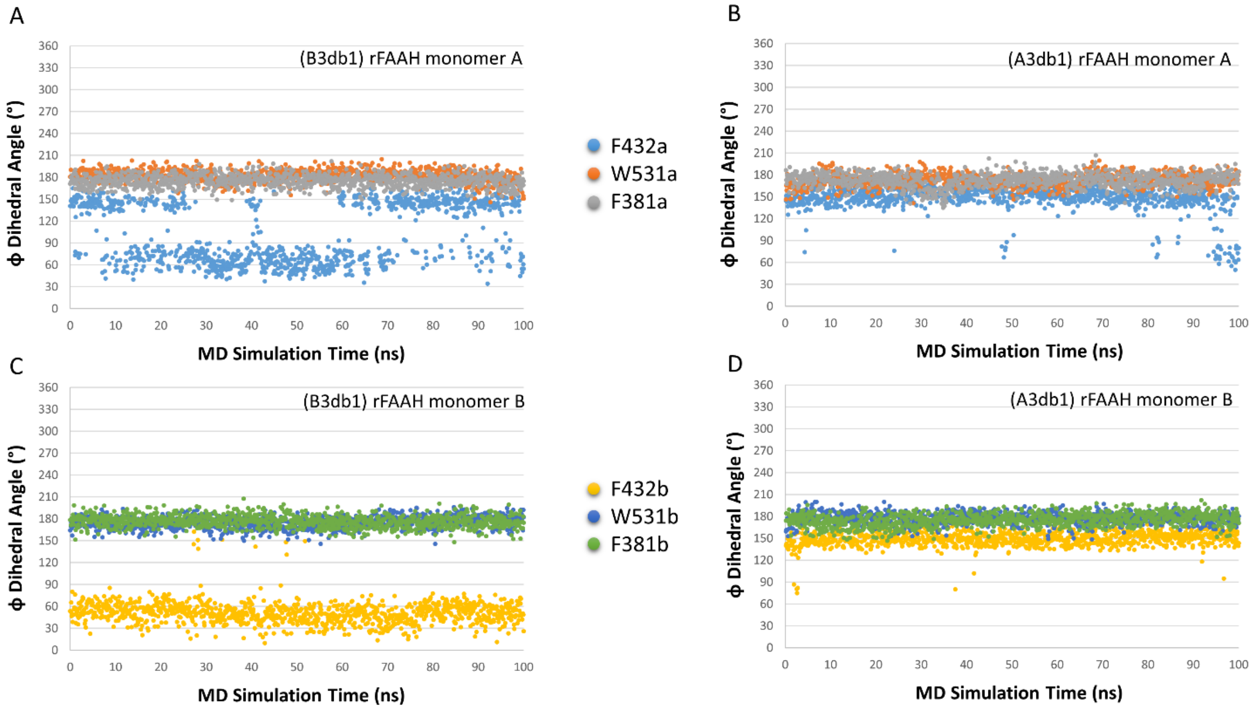

Figure S11. Plots of Cα-Cβ dihedral angles behaviour during 100 ns of MDs of the *"dynamic paddle"* forming residues F381 (gray and green points), F432 (cyan and yellow points) and W531 (orange and blue), in *r*FAAH monomers A and B, respectively. Plots were calculated from the starting docking pose of **TPA14** in the A3db1 and B3db1 binding modes.

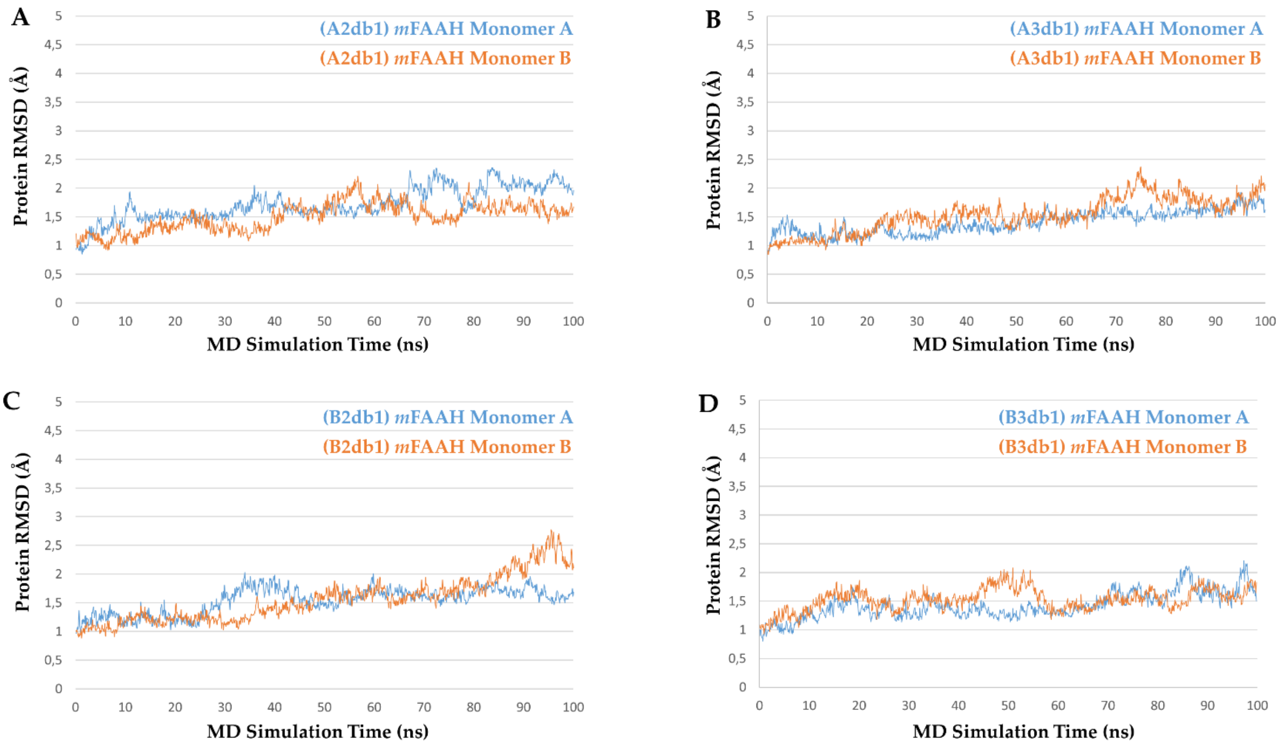

Figure S12. Protein RMSD during MD simulations of **TPA14** in *m*FAAH. Time evolution (ns) of the RMSD (Å) in monomer A (cyan) and monomer B (orange) of (A)A2db1; (B) B2db1; (C) A3db1; (D) B3db1.

Table S3. RMSD value (Å) of the ligand between clusters from each MDs of **TPA14** in *m*FAAH.

Pose	cluster	a0	a1	b0	b1	b2
**A2db1**	a0	/	N.A.[a]	2.6	1.4	N.A.
	b0	2.6	N.A.	/	2.2	N.A.
	b1	2.5	N.A.	2.2	/	N.A.
**A3db1**	a0	/	2.1	8.2	5.6	6.4
	a1	2.1	/	7.7	5.4	6.1
	b0	8.2	7.7	/	3.6	2.4
	b1	5.6	5.4	3.6	/	2.1
	b2	6.4	6.1	2.4	2.1	/
**B2db1**	a0	/	1.4	1.9	1.6	N.A.
	a1	1.4	/	1.8	1.4	N.A.
	b0	2	1.8	/	1.2	N.A.
	b1	1.6	1.4	1.2	/	N.A.
**B3db1**	a0	/	N.A.	5.5	4.7	N.A.
	b0	5.5	N.A.	/	2.8	N.A.
	b1	4.7	N.A.	2.8	/	N.A.

[a] Not Applicable. Poses not found.

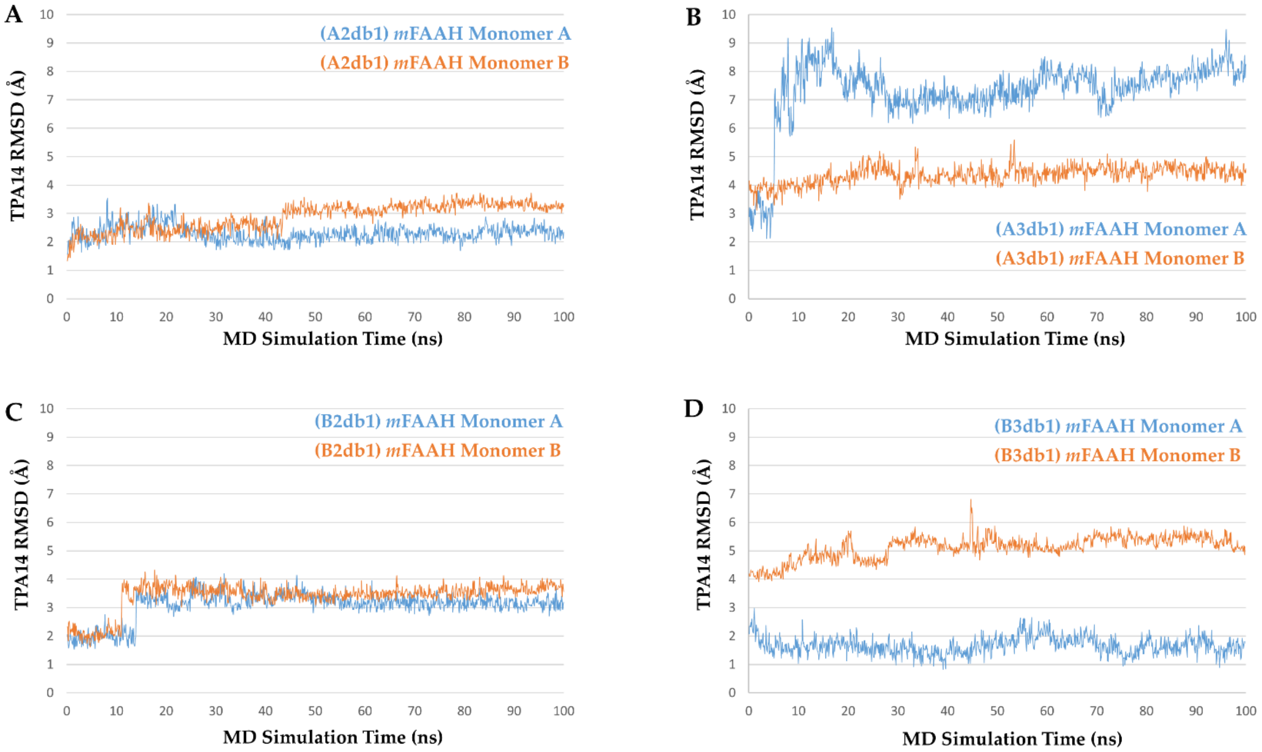

Figure S13. TPA14 RMSD during 100 ns of MDs simulations in *m*FAAH. Time evolution (ns) of the RMSD (Å) in monomer A (cyan line) and monomer B (orange line) of: (A) A2db1; (B) B2db1; (C) A3db1; (D) B3db1.

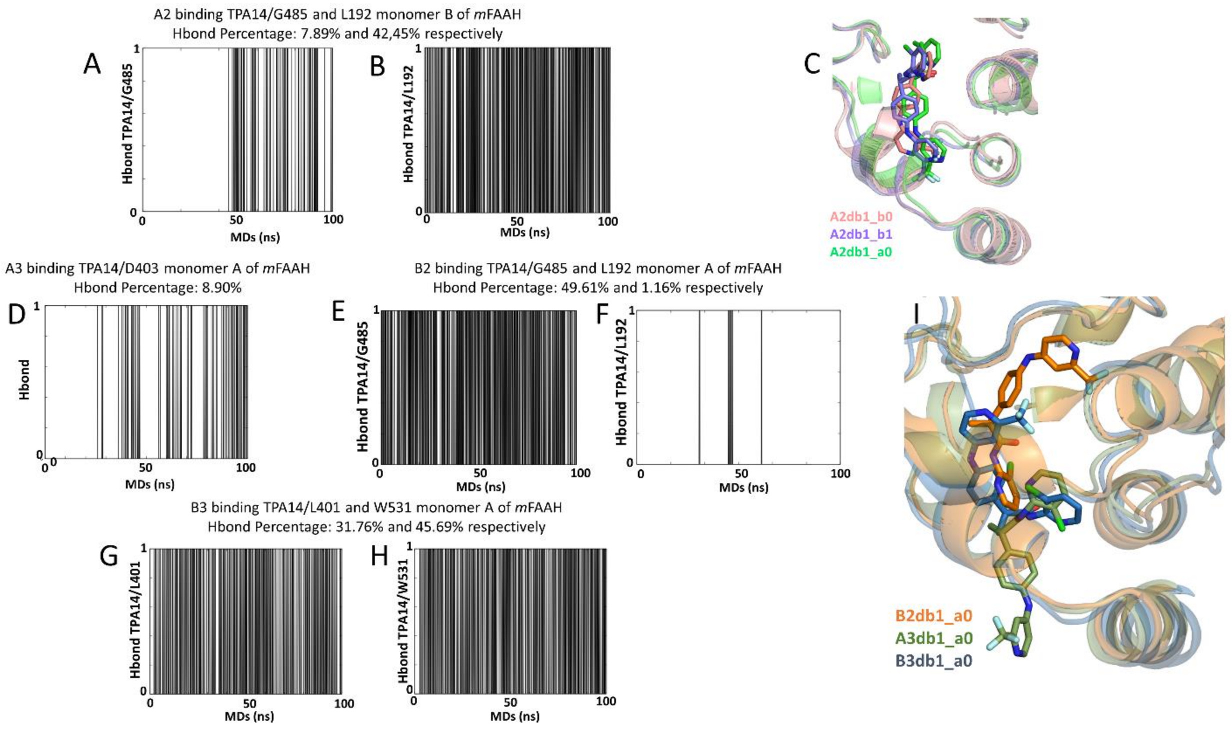

Figure S14: Structural differences between the TPA14 mFAAH clusters and the ligand/*m*FAAH key-interactions. (A-B) Key interactions of TPA14 in the A2db1 binding mode in mFAAH with G485 and L192; (C) Superposition among the A2db1 most populated clusters; (D-H) Key interactions of TPA14 in the A3db1, B2db1 and B3db1 binding modes of mFAAH with D403, G485, L192, L401 and W531 respectively; (I) Superposition of the most populated clusters in the monomer A of B2db1, A3db1 and B3db1 binding solutions.

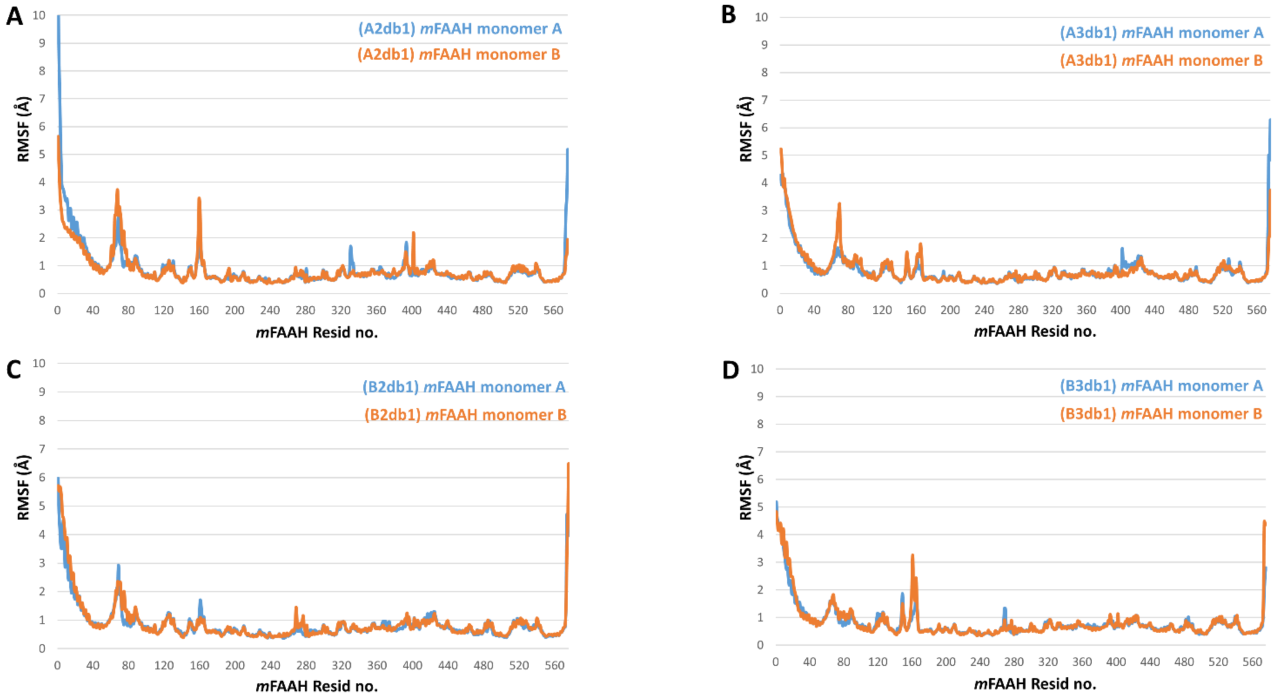

Figure S15. Root Mean Square Fluctuations (RMSF) of *m*FAAH in monomer A (cyan lines) and monomer B (orange lines) during 100 ns of MDs, calculated from the **TPA14** starting binding modes: (A) A2db1; (B) A3db1; (C) B2db1 and (D) B3db1.

References

1. Case, D. A.; Berryman, J. T.; Betz, R. M.; Cerutti, D.S.; Cheatham III, T. E.; Darden, T. A.; Duke, R. E.; Giese, T.J.; Gohlke, H.; Goetz, A. W.; Homeyer, N.; Izadi, S.; Janowski, P.; Kaus, J.; Kovalenko, A.; Lee, T. S.; LeGrand, S.; Li, P.; Luchko, T.; L, P.A. *Amber 2015*; University of California: San Francisco, **2015.**

2. Pastor, R.W.; Brooks, B.R.; Szabo, A. An Analysis of the Accuracy of Langevin and Molecular Dynamics Algorithms. Mol. Phys. **1988**, 65, 1409–1419.

3. Berendsen, H.J.C.; Postma, J.P.M.; van Gunsteren, W.F.; DiNola, A.; Haak, J.R. Molecular Dynamics with Coupling to an External Bath. *J. Chem. Phys.* **1984**, 81, 3684–3690.

4. Brown, P.M.; Steers, J.; Hui, S.W.; Yeagle, P.L.; Silvius, J.R. Role of Head Group Structure in the Phase Behavior of Amino Phospholipids. 2. Lamellar and Nonlamellar Phases of Unsaturated Phosphatidylethanolamine Analogues. *Biochemistry* **1986**, 25, 4259–4267.

5. Yeagle, P.L.; Bennett, M.; Lemaître, V.; Watts, A. Transmembrane Helices of Membrane Proteins May Flex to Satisfy Hydrophobic Mismatch. *Biochim. Biophys. Acta* **2007**, 1768, 530–537.
